# FGFR1–4 RNA-Based Gene Alteration and Expression Analysis in Squamous Non-Small Cell Lung Cancer

**DOI:** 10.3390/ijms231810506

**Published:** 2022-09-10

**Authors:** Joanna Moes-Sosnowska, Monika Skupinska, Urszula Lechowicz, Ewa Szczepulska-Wojcik, Paulina Skronska, Adriana Rozy, Aneta Stepniewska, Renata Langfort, Piotr Rudzinski, Tadeusz Orlowski, Delfina Popiel, Aleksandra Stanczak, Maciej Wieczorek, Joanna Chorostowska-Wynimko

**Affiliations:** 1 Department of Genetics and Clinical Immunology, National Institute of Tuberculosis and Lung Diseases, 01-138 Warsaw, Poland or; 2 Preclinical Development Department, Celon Pharma S.A, Research & Development Centre, 05-152 Kazun Nowy, Poland; 3 Department of Pathology, National Institute of Tuberculosis and Lung Diseases, 01-138 Warsaw, Poland; 4 Department of Surgery, National Institute of Tuberculosis and Lung Diseases, 01-138 Warsaw, Poland; 5 Clinical Development Department, Celon Pharma S.A., Research & Development Centre, 05-152 Kazun Nowy, Poland

**Keywords:** fibroblast growth factor receptor, FGFR1, FGFR2, FGFR3, FGFR4, gene expression, biomarkers, next-generation sequencing, NGS, Sq-NSCLC, squamous non-small cell lung cancer, targeted therapy, FGFR inhibitor

## Abstract

While fibroblast growth factor receptors (FGFRs) are involved in several biological pathways and FGFR inhibitors may be useful in the treatment of squamous non-small cell lung cancer (Sq-NSCLC), FGFR aberrations are not well characterized in Sq-NSCLC. We comprehensively evaluated FGFR expression, fusions, and variants in 40 fresh-frozen primary Sq-NSCLC (stage IA3–IV) samples and tumor-adjacent normal tissues using real-time PCR and next-generation sequencing (NGS). Protein expression of FGFR1–3 and amplification of *FGFR1* were also analyzed. *FGFR1* and *FGFR4* median gene expression was significantly (*p* < 0.001) decreased in tumors compared with normal tissue. Increased *FGFR3* expression enhanced the recurrence risk (hazard ratio 4.72, *p* = 0.029), while high *FGFR4* expression was associated with lymph node metastasis (*p* = 0.036). Enhanced *FGFR1* gene expression was correlated with FGFR1 protein overexpression (r = 0.75, *p* = 0.0003), but not with *FGFR1* amplification. NGS revealed known pathogenic *FGFR2,3* variants, an FGFR3::TACC3 fusion, and a novel TACC1::FGFR1 fusion together with *FGFR1,2* variants of uncertain significance not previously reported in Sq-NSCLC. These findings expand our knowledge of the Sq-NSCLC molecular background and show that combining different methods increases the rate of FGFR aberrations detection, which may improve patient selection for FGFRi treatment.

## 1. Introduction

Lung cancer is the most common cause of cancer-related death worldwide [1]. The squamous non-small cell lung cancer histotype (Sq-NSCLC) accounts for 20–30% of non-small cell lung cancer. The only approved novel first line systemic therapy for Sq-NSCLC is immune checkpoint inhibitors [2,3]. Therefore, it is important to identify effective targeted therapies and reliable predictive molecular biomarkers for Sq-NSCLC patients.

Recent studies have indicated the potential of fibroblast growth factor receptor inhibitors (FGFRis) as treatment due to the high rate of fibroblast growth factor receptor (FGFR) aberrations found in targetable oncogenic pathways [4] (reviewed in [5,6,7]). However, most early-phase clinical trials with FGFRis have shown only a partial response, which may be a result of the poor predictive power of FGFR1 amplification, which was initially the only predictive biomarker of response to FGFRis in Sq-NSCLC [7,8,9,10,11]. Subsequent studies revealed that FGFR mutations, fusions, or expression might provide more precise information on potential responders. In this context, *FGFR* gene variants and expression have been evaluated in several preclinical and clinical studies, albeit with conflicting results. For instance, *FGFR* mRNA or protein levels were reported as significant predictors of sensitivity to AZD4547 [12], BGJ398 [13], ponatinib [12,14], and rogaratinib [15], while others showed inconsistent results [10,16,17].

Members of the fibroblast growth factor (FGF) family, the *FGFR1–4* genes, encode four highly conserved tyrosine kinase receptors (FGFR1–4). Each FGFR consists of an extracellular region composed of three immunoglobulin-like domains (Ig I–Ig III), a single hydrophobic transmembrane domain, and a cytoplasmic tyrosine kinase domain. The extracellular domains interact with FGFs, which leads to dimerization of the FGFR followed by activation by sequential autophosphorylation of tyrosine residues [7]. FGFR signaling activates the phosphoinositide-3-kinase (PI3K)/AKT, signal transducer and activator of transcription (STAT), and mitogen activated protein kinase (MAPK) pathways [18]. Deregulated FGF/FGFR signaling through *FGFR* gene amplification, mRNA overexpression, mutation, or gene fusion is associated with ligand-independent dimerization of FGFRs and subsequent activation of cancer-related signaling pathways (PI3K/AKT, STAT, and MAPK), affecting cell proliferation, survival, metabolism, migration, and the cell cycle. While FGFR1–4 play roles in several biological pathways, simultaneous manifestation of their aberrations and expression in Sq-NSCLC has not been well characterized. Additionally, little is known about the clinical importance of FGFR1–4 expression. Finally, the difference between tumors and the surrounding lung tissue has not yet been comprehensively explored, especially in squamous lung cancer.

Therefore, to expand our knowledge of the role of FGFRs in Sq-NSCLC as well as their use as potential biomarkers for FGFRi treatment, a comprehensive evaluation of FGFR aberrations and their clinical importance in Sq-NSCLC was conducted using real-time PCR (RT-PCR) and next-generation sequencing (NGS) to assess gene expression, fusions, and variants, as well as FGFR1–3 protein expression and *FGFR1* amplification. Moreover, the difference between *FGFR1–4* expression levels in primary tumors and tumor-adjacent normal tissues, as well as the predictive value of *FGFR1–4* mRNA expression, was investigated.

## 2. Results

### 2.1. FGFR1–4 mRNA Expression in Sq-NSCLC Tumors and Tumor-Adjacent Normal Tissue

**Figure 1:**
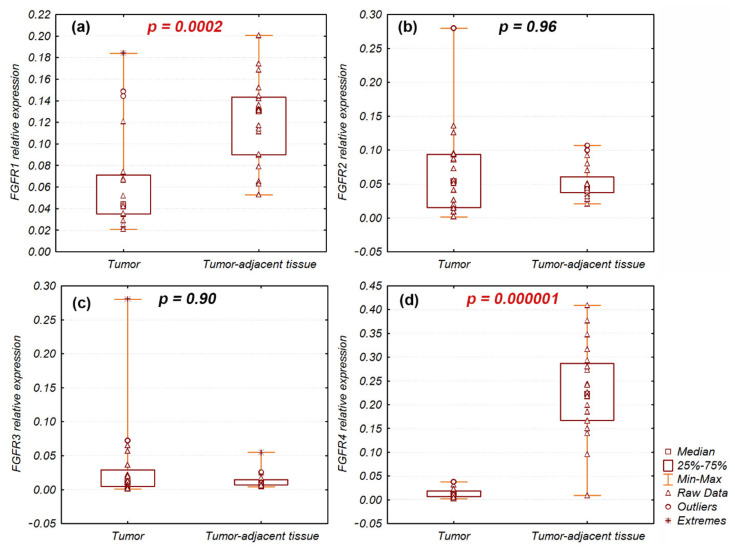
Relative expression of (**a**) *FGFR1*; (**b**) *FGFR2*; (**c**) *FGFR3*; (**d**) *FGFR4* genes in Sq-NSCLC tumor and tumor-adjacent normal tissues. Significant differences of *FGFR1* and *FGFR4* expression are indicated in red.

*FGFR1* and *FGFR4* gene expression levels were significantly decreased in tumor samples (n = 20) compared with tumor-adjacent normal tissue (n = 20): *FGFR1* (*p* = 0.0002) and *FGFR4* (*p* = 0.000001) (Figure 1). Nevertheless, *FGFR1* and *FGFR4* expression levels were increased and approximated to expression in tumor-adjacent normal tissue (fold-change, ~1) in individual tumor samples: 3 (15%) and 1 (5%), respectively (Figure S1). *FGFR2* and *FGFR3* mRNA expression levels were not significantly different between tumor and tumor-adjacent normal tissues (*p* = 0.97 and *p* = 0.9, respectively). However, in individual tumor samples, *FGFR2* and *FGFR3* expression levels were enhanced: fold-changes > 2 were observed in 5 (12.5%) and 7 (17.5%) samples, respectively (Figure S1).

**Figure 2:**
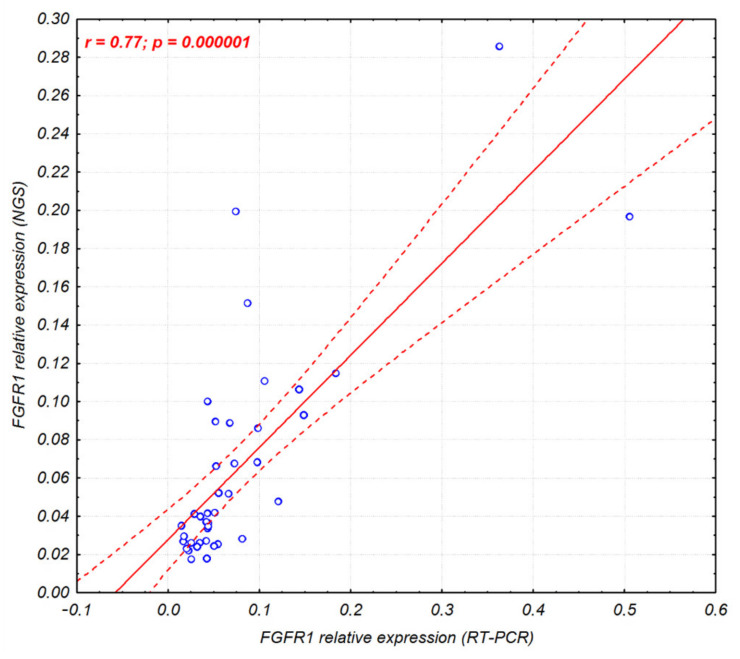
Correlation scatter plot of the *FGFR1* gene expression assessed by RT-PCR and NGS (Archer Lung FusionPlex) in 40 Sq-NSCLC tumor samples. Corresponding figures for *FGFR2* and *FGFR3* gene expression are shown in Supplementary Figure S2.

Additionally, since the anchored multiplex PCR followed by NGS can be used in gene expression studies, the *FGFR1-3* expression levels obtained from the RT-PCR and NGS were compared. The analysis revealed high correlation within *FGFR1*, *FGFR2*, and *FGFR3* expression assessed with both methods (r = 0.77, *p* = 0.000001; r = 0.86, *p* = 0.000001; and r = 0.95, *p* = 0.000001, respectively; Figure 2 and Supplementary Figure S2) in 40 Sq-NSCLC tumor samples.

### 2.2. Clinical Significance of FGFR1–4 mRNA Expression 

**Figure 3:**
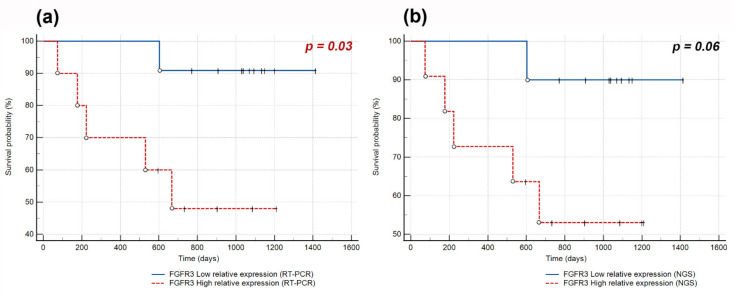
Kaplan–Meier curves of disease-free survival (DFS) obtained by stratifying Sq-NSCLC patients with follow-up data available according to *FGFR3* expression level assessed by (**a**) RT-PCR with a cut-off point at the median value of 0.021; (**b**) NGS with a cut-off point at the median value of 0.023. The complete observations are indicated by circles and censored by vertical marks.

Analysis of *FGFR1–4* expression and disease-free survival (DFS) revealed that the increased level of *FGFR3* mRNA was correlated with an increased risk of recurrence (RT-PCR: hazard ratio (HR) 4.72, *p* = 0.029; NGS: HR 7.9, *p* = 0.0049). Kaplan–Meier survival curves also showed a trend toward poorer prognosis for patients with high *FGFR3* expression compared to those with low expression (Figure 3). The mean DFS time of patients with high and low *FGFR3* expression was 620 days (<2 years) and 1037 days (~3 years), respectively. *FGFR1* (RT-PCR: *p* = 0.44, NGS: *p* = 0.56), *FGFR2* (RT-PCR: *p* = 0.22, NGS: *p* = 0.19), and *FGFR4* (*p* = 0.97) expression levels did not affect the risk of recurrence.

### 2.3. Association between the FGFR1–4 Expression and Clinicopathological Features

**Figure 4:**
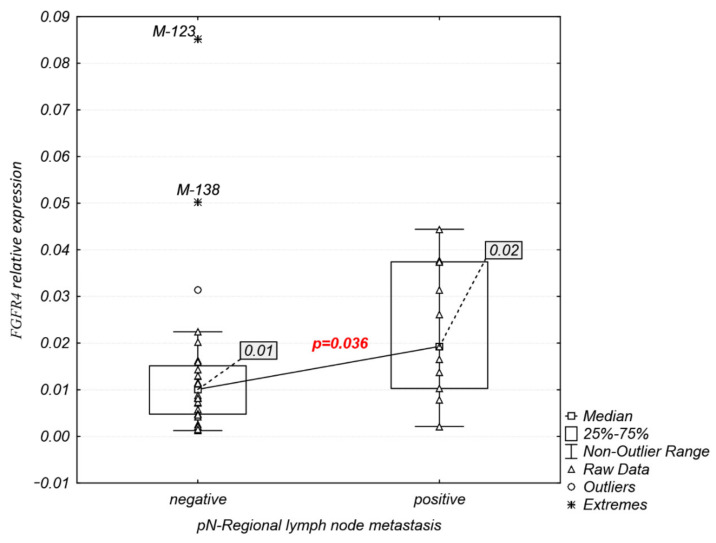
Significant associations of *FGFR4* expression with pN-regional lymph node metastasis.

Interestingly, analysis of *FGFR1–4* expression association with clinicopathological characteristics (Table 1) revealed that the *FGFR4* mRNA expression was significantly higher in patients with lymph node metastasis (median: 0.02 vs. 0.01, *p* = 0.036; Figure 4), while *FGFR1–3* expression levels were not significantly related to any of the clinicopathological features examined. 

### 2.4. Analysis of FGFR1–3 Protein Overexpression and FGFR1 Amplification

**Figure 5:**
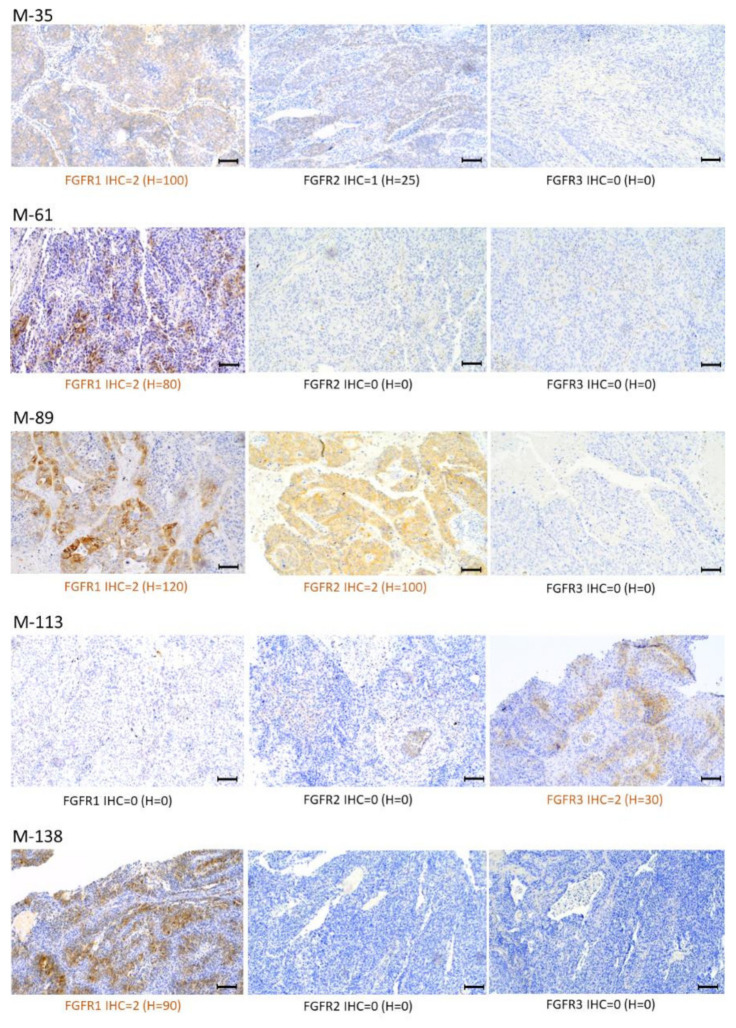
Patterns of FGFR1, FGFR2, and FGFR3 expression via immunohistochemistry (IHC) in selected Sq-NSCLC samples (magnification, ×100; scale bars, 100 μm). Staining intensity (described in the Materials and Methods) was stratified according to: a four-graded scale: negative (IHC = 0), weak (IHC = 1), moderate (IHC = 2), and strong (IHC = 3); and H-score determined as follows: 0 x (% cells with no staining [0]) + 1 × (% cells staining faint, weakly [1+]) + 2 × (% cells staining moderately [2+]) + 3 × (% cells staining strongly [3+]).

**Figure 6:**
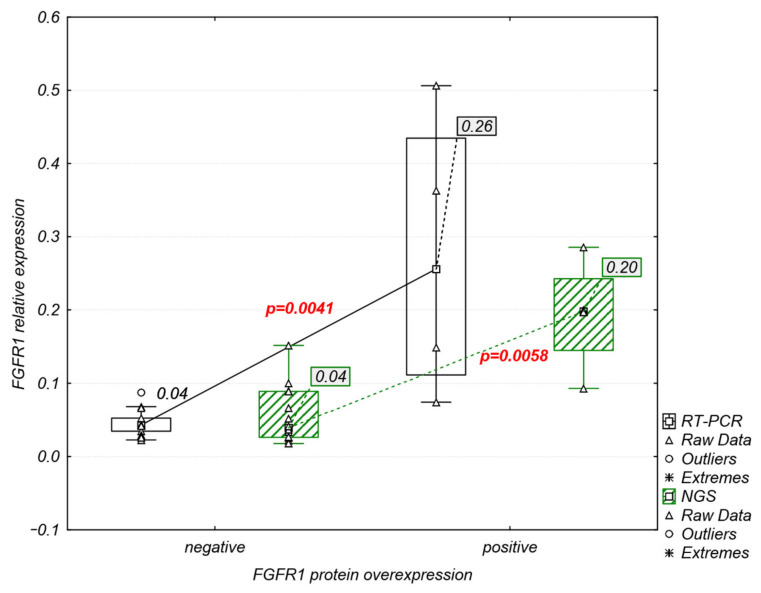
Significant association between relative mRNA and protein levels of *FGFR1*.

FGFR1–3 protein overexpression was detected both in the cytoplasm and cell membrane in 5 of 18 (27.7%) tumor samples (Figure 5). FGFR1 overexpression was observed in four samples (M-35, M-61, M-89, and M-138), while one sample showed co-expression of FGFR1 and FGFR2 (M-89). FGFR3 protein overexpression was observed in one sample (M-113) (Figure 5). Comparative analysis of *FGFRs* mRNA and protein expression revealed that FGFR1 protein overexpression was significantly correlated with increased mRNA levels of *FGFR1* (RT-PCR: r = 0.75, *p* = 0.0003; NGS: r = 0.67, *p* = 0.002) (Figure 6).

**Figure 7:**
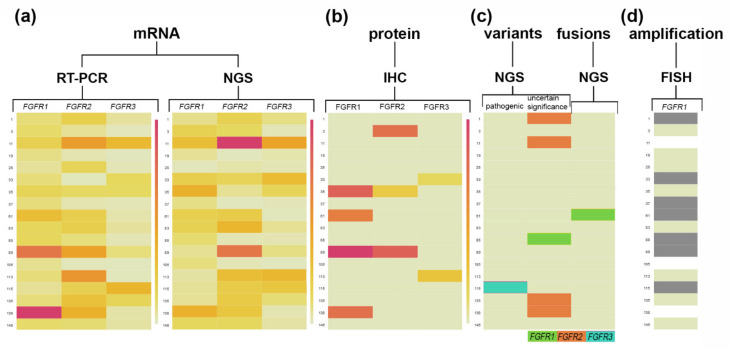
FGFR1, FGFR2, and FGFR3 transcript and protein levels together with *FGFRs* variants and *FGFR1* amplification status. Heat maps illustrate the (**a**) *FGFR1-3* relative gene expression values (mRNA) with differentiation for RT-PCR and NGS methods; (**b**) FGFR1-3 protein expression levels based on H-score; (**c**) *FGFRs* variants occurrence with differentiation for clinical significance and FGFRs fusions; (**d**) *FGFR1* amplification status based on FGFR1/CEN8 ≥ 2.0 or the average number of *FGFR1* signals per cell ≥ 6 or ≥10% of tumor cells containing ≥ 15 *FGFR1* signals or large clusters; for Sq-NSCLC samples. The largest gene expression values (**a**,**b**) are displayed in red color, intermediate values in shades of orange and yellow, and the smallest values in light yellow. Dark colored cells in the map (**c**,**d**) represent the variants, fusion, or an *FGFR1* amplification occurrence, while no *FGFRs* variants, fusion, or amplification occurrence is indicated in light yellow. Not analyzed cells are indicated in white.

*FGFR1* amplification was detected in 7 of 15 (46%) samples with available fluorescence in situ hybridization (FISH) results (Figure 7), while high *FGFR1* amplification was observed in three samples (20%) (M-37, M-61, and M-89). In two samples (M-61 and M-89), concurrent FGFR1 amplification and protein was observed. Statistical analysis revealed no correlation between *FGFR1* amplification and mRNA (RT-PCR: *p* = 0.58, NGS: *p* = 0.37) or protein (*p* = 0.47) expression levels.

### 2.5. Detection of FGFR Oncogenic Gene Fusions and Genetic Variants

#### 2.5.1. Squamous Non-Small Lung Tumors

RNA analysis was performed for all 40 tumor and five tumor-adjacent normal samples. In the tumor group, two (5%) different (“strong” and “low confidence”) FGFR gene fusions were detected (Table 2). The first identified fusion was previously known as the FGFR3::TACC3 fusion (ex17::ex11) (M-20; Figure S3). Its exact breakpoint is indicated in the Archer Quiver database, and despite the low percent structural variation reads for GSP2 (5.49%) was categorized as “strong”. The second discovered fusion event was the TACC1::FGFR1 fusion (ex2::ex2) (M-61; Figure S4), which was categorized as “low confidence” because of the low percent of structural variation reads for GSP2 (4.66%). No fusion events were found in tumor-adjacent normal tissues (M-20P, M-33P, M-61P, M-135P, and M-138P).

*FGFR* variant analysis (based on the ClinVar database) revealed two pathogenic variants (5%), three variants of uncertain significance (7.5%), and one variant with no clinically significant information (2.5%) (Table 2). Pathogenic, missense variants of *FGFR2* (c.870G>T), and *FGFR3* (c.746C>G) were detected in two individual tumors (M-119 and M-115, respectively). Among the variants of uncertain significance, the missense *FGFR1* (c.899T>C, p.(Ile300Thr)) and *FGFR2* (c.2419G>A, p.(Glu807Lys)) gene variants were found in single tumors (M-88 and M-1, respectively), while the frameshift variant of *FGFR2* (c.2398dup, p.(Ser800PhefsTer22)) was found in seven tumors and one tumor-adjacent normal tissue sample. The missense variant of *FGFR2* (c.2211G>T, p.(Met737Ile)) with no clinical significance in the ClinVar database was detected in one tumor (M-6).

Correlations of described *FGFRs* genetic changes with *FGFRs* expression level in Sq-NSCLC revealed statistically significant but weak association of *FGFR2* c.2398dup (p.(Ser800PhefsTer22)) variant with the increased *FGFR2* mRNA expression level (*p* = 0.02, r = 0.36).

#### 2.5.2. Commercial Controls

We detected 12 gene fusions and 2 oncogenic isoforms (100%) in the commercially available positive control (Seraseq) with the Archer FusionPlex Lung gene NGS panel (Table S1). Thirteen (92.85%) met all basic recommendations for “strong” fusion detection: number of start sites over 3, minimum of 5 unique breakpoint-spanning reads that support the gene fusion, and minimum of 10% of total reads for GSP2 over the wild-type transcript. The only remaining fusion, SLC45A3::BRAF, had 3.67% of reads supporting the fusion, below the default (10.0%) threshold. The negative control for EML4::ALK, CCDC6::RET, SLC34A2::ROS1, TPM3::NTRK1, and ETV6::NTRK3 fusions (Horizon) was identified as fusion-negative (100%) using the NGS panel.

## 3. Discussion

Our findings revealed significantly decreased *FGFR1* and *FGFR4* mRNA expression in tumor tissue compared with tumor-adjacent normal tissue. Meanwhile, expressions of both genes were enhanced in selected tumor samples, reaching values similar to surrounding normal tissue. *FGFR2* and *FGFR3* mRNA expression varied between tumor and tumor-adjacent normal tissues and were enhanced 2–10 times in several tumor samples. Accordingly, *FGFR1* mRNA expression was lower in Sq-NCLC compared with normal tissues as per The Cancer Genome Atlas and Genotype-Tissue Expression data [19]. FGFR1 expression has recently been linked to the highly negative correlation between *FGFR1* mRNA and methylation levels (average of three CpG sites: cg10823844, cg15791248, and cg27646230) in tumor samples [19]. In contrast to our results, Ren et al. [13] demonstrated that nearly 50% of tumors had an increase in *FGFR1* mRNA expression compared with tumor-adjacent normal tissue; however, in their study, *FGFR1* expression was normalized to *GAPDH*. Research has indicated that the choice of stable reference gene is crucial for accurate results in gene expression studies [20,21,22]. Our previous results assessing reference genes in Sq-NSCLC showed that *GAPDH* gene expression was diverse and unstable in tumor and tumor-adjacent normal tissue samples, whereas *POLR2A* and *ACTB* expression levels were the most stable among analyzed samples (Figure S5) [22]. 

To our knowledge, this study is the first to show significantly lower *FGFR4* mRNA expression in Sq-NSCLC compared with normal lung tissue. Huang et al. [23] as the only one compared *FGFR4* mRNA expression levels in lung tumors and adjacent normal tissues with results inconsistent with our results, i.e., the *FGFR4* mRNA level was significantly higher in tumor tissues. Importantly, in that study [23], the detailed tumor histotype was not mentioned and *GAPDH* was used for normalization. Additionally, lower *FGFR4* expression at the mRNA and protein levels was reported in lung tissues obtained from patients with idiopathic pulmonary fibrosis (IPF) compared with control patients. The authors indicated that the lower FGFR expression level was related to FGFR4 downregulation by pro-fibrotic factors (TGFβ, CTGF, and ET-1) [24]. Simultaneously, our work revealed enhanced *FGFR4* mRNA levels in several samples. In vitro analysis of squamous lung cancer cell lines showed that *FGFR4* overexpression leads to FGFR4 auto-activation and increased cell growth, clonogenicity, soft agar colony formation [25], and enhanced cell proliferation, whereas knockdown of *FGFR4* can reduce proliferation [23]. Recent studies have indicated that FGFR4 deficiency might regulate the tumor immune microenvironment by activating the antigen presentation process and cellular immunity to the change in sensitivity to immune checkpoint inhibitor treatment in NSCLC [26]. 

In the present study, we revealed a significant association between increased *FGFR4* expression and lymph node metastasis in Sq-NSCLC, but there was no significant association between *FGFR1–4* mRNA expression and other clinicopathological features. A relationship between FGFR4 and lymph node metastasis has been implied. High FGFR4 protein overexpression has been correlated with lymph node metastasis in triple-negative breast cancers [27] and gastric cancer [28,29]. Earlier studies demonstrated that FGFR4 overexpression may be a result of gene amplification, especially in breast cancer tumors with high lymph node metastases, as well as in estrogen receptor- and progesterone receptor-positive tumors [30]. Additionally, the *FGFR4* p.(Gly388Arg) variant, located in the transmembrane domain, has been correlated with poorer overall and progression-free survival in Sq-NSCLC patients with lymph node involvement [31]; this can be linked to increased FGFR4 stability and sustained activation, as has been shown in prostate cancer [32]. 

Herein, we present the novel finding that increased *FGFR3* mRNA expression might be a negative prognostic marker in terms of the risk of recurrence of squamous cell lung cancer. Due to the relatively small number of patients analyzed, our results should be interpreted with caution. Nevertheless, mRNA levels of *FGFR3* mRNA have been associated with worse DFS in oropharyngeal squamous cell carcinoma (*p* = 0.005) [33] and in non-muscle-invasive bladder cancer (HR 3.78, *p* < 0.001) [34]. The *FGFR3* mRNA level is also a negative prognostic factor for lung adenocarcinoma [35] and squamous cell laryngeal cancer [36], where high *FGFR3* expression was significantly correlated with shorter overall survival (OS). By contrast, high *FGFR3* mRNA expression was associated with better progression-free survival in patients with primary pT1 bladder cancer (log-rank, *p* < 0.001) [37]. Our study did not reveal any clinical significance of *FGFR1, 2*, and *4* mRNA levels in terms of tumor relapse; however, the impact of *FGFR1* mRNA level on squamous cell lung cancer patient survival remains controversial. For instance, elevated FGFR1 expression was reported as a negative factor that negatively impacted patient survival [38], while Wynes et al. [14] revealed no prognostic association with OS or DFS. OS is a widely used endpoint for assessing the prognostic value of studied variables. However, censoring patients at death or the date of last follow-up (for OS), especially in groups with a small number of observations, can lead to an overestimation of OS [39]. In the present study, only DFS (i.e., the time to cancer recurrence or death from any cause) was analyzed. 

Additionally, the presented results show the usefulness of anchored multiplex PCR followed by NGS for *FGFR1–3* gene expression analysis. The high correlation (77–95%) of *FGFR* expression obtained with the use of RT-PCR and an NGS panel was observed, despite the different sets of reference genes. In RT-PCR, *POLR2A* and *ACTB* were used to normalize expression, while the NGS panel contained *CHMP2A*, *GPI*, *RAB7A*, and *VCP*. Thus far, use of the relative gene expression data obtained with the applied NGS panel for genetic profiling has been described for acute myeloid leukemia [40], while it was previously limited to determining gene fusion occurrence [41,42]. 

Our study confirmed that FGFR1 protein overexpression is more frequent (6–9%) [19,43] than overexpression of FGFR2 and FGFR3 (3.4% and 6.6% [44]) in Sq-NSCLC. Moreover, in agreement with Rooney et al. [12] and Bogatyrova et al. [19], we confirmed the significant association between *FGFR1* mRNA and protein overexpression. Remarkably, there was no significant correlation between *FGFR1* mRNA/protein expression and gene amplification, likely due to the fact that only 31–50% of Sq-NSCLCs with an increased *FGFR1* gene copy number overexpressed *FGFR1* mRNA, while *FGFR1* mRNA expression was absent in 25% of *FGFR1*-amplified tumors [4,14,45]. 

Considering that *FGFR* mutations and fusions are detected in Sq-NSCLC (reviewed in [7]), we performed targeted *FGFR* sequencing with the use of anchored multiplex PCR technology followed by NGS, which was previously shown to efficiently discover genomic aberrations, including novel fusions. We found one FGFR3::TACC3 fusion (2.5%), which is the most frequent somatic translocation in Sq-NSCLC (range: 0.6–5.3%) [44,46,47,48,49,50,51,52,53]. Interestingly, mRNA expression of *FGFR3* was slightly increased (fold change = 1.5) compared with tumor-adjacent normal tissue in this sample. Nonetheless, previous reports have shown that this fusion event is not correlated with *FGFR3* mRNA or protein overexpression in Sq-NSCLC [44], in contrast with glioblastomas [54]. Parker et al. [55] suggested that loss of the 3′ region of *FGFR3* might abolish downregulation by miR-99a and lead to overexpression of the fusion gene. We also detected a new somatic fusion event, a combination of *TACC1* and *FGFR1*. Truncated TACC1 was fused before the FGFR1 extracellular immunoglobulin-like domain (Ig). Because this fusion did not cause truncation of FGFR1, its effect remains unknown. Additionally, *FGFR1* amplification together with mRNA and protein overexpression were observed in this tumor. Two pathogenic, missense variants of *FGFR2* and *FGFR3*, c.870G>T (p.(Trp290Cys)) and c.746C>G (p.(Ser249Cys)), were also detected at a frequency of 5%, consistent with previously published results [8,10,56,57,58,59,60]. Both variants are located in the FGFR protein extracellular domain (Ig III) and induce constitutive dimerization and receptor activation via modest dimer stabilization in the absence of ligand [61]. Among variants of uncertain significance (based on the ClinVar database), we identified the *FGFR1* (c.899T>C, p.(Ile300Thr)) and *FGFR2* (c.2419G>A, p.(Glu807Lys)) missense variants and one *FGFR2* frameshift (1 bp duplication) variant (c.2398dup, p.(Ser800PhefsTer22)). To our knowledge, this is the first detection of these variants in Sq-NSCLC. The *FGFR1* (c.899T>C) missense variant was previously reported in craniosynostosis by Wilkie et al. [62] and nonsyndromic trigonocephaly by Kress et al. [63]. This variant results in amino acid substitution p.(Ile300Thr) located in the extracellular domain (Ig III). However, based on *in silico* analysis (UniProt) available via the VarSome database, it is unclear whether this variant causes a pathogenic or benign effect on the protein. Clinical significance scoring (based on the American College of Medical Genetics (ACMG) guidelines for the interpretation of sequence variants [64]) showed that it is likely a benign variant. The *FGFR2* (c.2419G>A) variant results in an amino acid substitution at the end of the cytoplasmic domain of FGFR2 (p.(Glu807Lys)), aside from the kinase domain. Localization of this variant indicates a weak clinical relevance, confirmed by VarSome database scoring, suggesting a benign clinical significance. Until now, occurrence of this variant has been associated with Apert syndrome (acrocephalo-syndactyly type 1) in the ClinVar database. No further information or publications are available. The last detected variant of uncertain significance, *FGFR2* (c.2398dup), results in a premature translational stop codon in the *FGFR2* gene (p.(Ser800Phefs*22)). CMG classification in the VarSome database indicates a pathogenic clinical significance. Our results showed slightly association with increased *FGFR2* mRNA expression level, but without protein overexpression what may arise from the issue that the frameshift is located at the end of the cytoplasmic domain of the FGFR2 protein, next to the kinase domain, and results in disruption of the last 23 amino acids of the FGFR2 protein. Therefore, its clinical relevance may be weak. This variant was also detected in tumor-adjacent normal tissue, indicating germline origin. It has not been reported previously in the literature (with the exception of the ClinVar database where it was linked with craniosynostosis), which might be a result of its low allelic frequency (~2.5%). We also report a new, single-nucleotide variant of the *FGFR2* gene (c.2211G>T), which causes a missense amino acid change p.(Met737Ile) at the end of the kinase domain. ACMG classification in the VarSome database indicates a likely pathogenic effect; there is no available information on its functional and clinical significance.

Finally, presented results revealed *FGFR* overexpression, variants, and fusions considered as potential biomarkers of the response to FGFRi treatment. For instance, enhanced *FGFR1–3* mRNA expression levels represent promising FGFRi biomarkers since 5.6% of patients treated with rogaratinib (NCT03762122) achieved partial response and 64% had stable disease [65]. Interestingly, the FGFR3::TACC3 fusion is considered an FGFRi predictive biomarker in gliomas (NCT02824133, NCT04424966) and cholangiocarcinoma (NCT03773302) [66]. However, in Sq-NSCLC, few clinical studies, and a scarcity of available data, mostly from individual Sq-NSCLC cases [9,67], indicate the need for further research in this field. Clinical studies with AZD4547 (SWOG S1400D, NCT02965378, NCT00979134) revealed Sq-NSCLC patients with *FGFR3* p.(Ser249Cys) who achieved partial response (1.5 months with ~32% tumor shrinkage) [9] or stable disease (2.6-month progression-free survival and 12% tumor shrinkage) [10], while no significant benefit of AZD4547 [9] or BGJ398 (NCT01004224) [8] was shown in two other patients (2–4% tumor size decrease). Our results also identify *FGFR1* and *FGFR2* variants not previously described in Sq-NSCLC; the predictive power of these variants for FGFRi treatment will require further investigation.

## 4. Materials and Methods

### 4.1. Patient and Tumor Selection

This study included patients with a lung cancer diagnosis (n = 63) (Sq-NSCLC (n = 56) and adenosquamous carcinoma (n = 7)) who underwent a surgical procedure at the National Institute of Tuberculosis and Lung Diseases and for whom cancer-tissue samples (formalin-fixed, paraffin-embedded (FFPE)) were obtained during the intraoperative procedure performed for diagnostic purposes. Tumor tissue samples, together with corresponding tumor-adjacent normal tissue (taken from the surgical resection margin at least 5 cm from the tumor), were snap-frozen in liquid nitrogen followed by storage at −80 °C until further analysis (“fresh-frozen” tissue samples). Cryostat sections were stained with hematoxylin and eosin and evaluated by a pathologist for cancer cell content, stromal cell contamination, and necrosis. Only tumor samples from 46 patients containing greater than 50% cancer cells and tumor-adjacent normal tissue samples without any cancer cells were selected.

Finally, the study group consisted of 40 cancer patients for whom a sufficient amount of RNA was available. All tumors were uniformly reviewed and classified histologically according to the World Health Organization guidelines [68]. Clinical stage was determined with the use of the TNM Classification of Malignant Tumors (8th edition) [69]. Clinicopathological characteristics are presented in Table 1.

### 4.2. RNA Control Material for NGS

Two commercially available reference samples for fusion detection, positive control-Seraseq® FFPE Tumor Fusion RNA Reference Material v2 (SeraCare, Milford, MA, USA) and negative control-5 Fusion Multiplex RNA Negative Control (Horizon Discovery, Waterbeach, UK), were used. The Seraseq material contained 12 significant RNA fusions and two oncogenic isoforms that could be detected with the Lung FusionPlex panel (Table S1). The Horizon material was negative for five common gene fusions (EML4::ALK, CCDC6::RET, SLC34A2::ROS1, TPM3::NTRK1, and ETV6::NTRK3).

### 4.3. RNA Extraction and cDNA Synthesis 

Total RNA was extracted from fresh-frozen tissues with the use of RNeasy Plus Mini Kit (Qiagen, Hilden, Germany) according to the manufacturer’s instructions. gDNA Eliminator columns allowed for the elimination of genomic DNA while avoiding RNA damage during DNase digestion. RNA quantity (260/280 ratio) was measured with the use of a NanoDrop UV spectrophotometer (ThermoFisher, Waltham, MA, USA) and Quantus fluorometer (Promega, Madison, WI, USA). Additionally, RNA quality was assessed by RNA electrophoresis with the 2100 Bioanalyzer System (Agilent, Santa Clara, CA, USA). The RNA integrity number ranged from 4.4 to 9.8 with a median value of 8.8. One microgram of total RNA was transcribed to cDNA using the High-Capacity cDNA Reverse Transcription Kit (ThermoFisher) with random primers according to the manufacturer’s instructions.

### 4.4. Real-Time Polymerase Chain Reactions (RT-PCR)

RT-PCR was run in triplicate using the TaqMan Universal PCR Master Mix with AmpErase™ Uracil N-Glycosylase (ThermoFisher) and approximately 10 ng of total RNA from 40 tumors and 20 corresponding tumor-adjacent normal tissue samples reverse transcribed to cDNA. Quantitative RT-PCR was run on the 7500 Fast Real-Time PCR System (Applied Biosystems, Waltham, MA, USA) with the use of the FAM- and VIC-labeled TaqMan Gene Expression Assays (ThermoFisher) for *FGFR1* (Hs00241111_m1), *FGFR2* (Hs01552918_m1), *FGFR3* (Hs00179829_m1), *FGFR4* (Hs00242558_m1), *POLR2A* (Hs00172187_m1), and *ACTB* (Hs99999903_m1). The RT-PCR results were averaged and *FGFR1–4* gene expression levels were normalized to the reference genes *ACTB* and *POLR2A* (Figure S5) [22]. Gene expression was analyzed with the use of a relative quantification method. For the 20 patients with both tumor and corresponding tumor-adjacent normal tissue samples available, data were expressed as a fold-change in expression between tumor and normal samples (−2^−ΔΔCt^ method).

### 4.5. Next-Generation Sequencing (NGS)

RNA from tumor tissue and tumor-adjacent normal tissue was sequenced and screened for gene fusions, variants, and expression of 14 genes of interest (*ALK, BRAF, EGFR, FGFR1–3, KRAS, MET, NRG1, NTRK1–3, RET*, and *ROS1*) using the FusionPlex Lung kit (Archer Dx, Boulder, CO, USA). Briefly, RNA (68–250 ng) was transcribed to cDNA using random priming. Next, cDNA quality was checked with the PreSeq RNA QC Assay (Archer Dx). Only cDNA with PreSeq result CP < 28 was used for DNA library construction according to the manufacturer’s instructions. Subsequently, concentration and quality of obtained libraries were determined using the KAPA Universal Library Quantification Kit (Roche Diagnostics, Basel, Switzerland). Next, libraries were normalized, multiplexed, and sequenced using the MiSeq Reagent Kit, v3 (600 cycles) (Illumina, San Diego, CA, USA) on the MiSeq platform. Samples were sequenced in four runs with average quality parameters: QC30 of 85.4% and cluster density of 1324 k/mm^2^. The Illumina MiSeq sequencer generated paired-end sequence reads with an average of 1.15 million (range: 0.38–3.5) reads per sample (detailed sample statistics are shown in Table S2). NGS results were analyzed in Archer Analysis software v6.2 (Archer Dx), which aligns sequencing data against the human genome (version hg19) with the use of BWA (Burrows-Wheeler Alignment Tool) and Bowtie 2 (an ultrafast, memory-efficient short read aligner) for mapping. For the quality check (QC metric), four control genes (*CHMP2A* (charged multivesicular body protein 2A), *GPI* (glucose-6-phosphate isomerase), *RAB7A* (RAB7A, member RAS oncogene family), and *VCP* (valosin containing protein)) served as reliable indicators of overall RNA quality and content in the sample. A QC metric of at least 10 was required to support the targets of the assay. The median QC metric of analyzed samples was 503.88 (range: 242.5–607.4). 

Gene fusions were called with the following detection limits: total number of supportive reads spanning the fusion junction ≥ 5; number of unique start sites for the fusion sequence specific primer ≥ 3; and percent of supporting reads at breakpoint supporting fusion ≥ 10%. Analysis of a control sample (described below) revealed that the percent of reads at breakpoint supporting fusion could be lowered to 3.6%. Results of the fusion annotation were split into two categories: “strong confidence” fusion and oncogenic isoform candidates and “low confidence” fusion candidates. 

Gene variants were called and listed when the altered allele frequency was ≥5%, altered allele count was ≥10%, and read depth was ≥100 reads. Additionally, detected variants with an allele frequency above 2% with a minimum sequencing depth of ≥100 reads and a minimum variant depth of 10% were kept and listed if they were found in ClinVar or COSMIC databases, or had a deleterious impact on the protein. All detected variants were reviewed manually with the use of Archer Analysis software. 

The relative gene expression level was assessed based on the ratio of averaged unique RNA reads originating from all GSP2 primers across the targeted and housekeeping genes (*CHMP2A*, *GPI*, *RAB7A*, and *VCP*) with Archer Analysis software.

### 4.6. Immunohistochemistry (IHC)

FGFR1, FGFR2, and FGFR3 protein expression levels were determined in 18 tumors (FFPE tissue samples). Immunohistochemistry (IHC) was performed according to the manufacturer’s instructions. Slides were stained with the Dako Omnis immunostainer and Dako EnVision Flex + reagents (Dako Omnis, Dako Agilent Technologies, Leuven, Belgium). Tissues were incubated with the following primary antibodies: anti-FGFR1 (clone D8E4, Cell Signaling Technology, Danvers, MA, USA), anti-FGFR2 (ab10647, Abcam, Cambridge, UK), and anti-FGFR3 (clone B-9; Santa Cruz Biotechnology, Santa Cruz, CA, USA) as previously described [70,71,72]. The IHC slides were counterstained with hematoxylin and coverslips were applied. Staining intensity was categorized based on a four-level scale: negative (0), weak (1), moderate (2), and strong (3). Overexpression was defined as follows: FGFR1—moderate (2) or strong (3) intensity membrane and/or cytoplasmic staining in ≥ 10% tumor cancer cells previously described by Theelen et al. [44]; FGFR2—moderate (2) or strong (3) complete membrane staining intensity in ≥ 10% tumor cancer cells evaluated according to the HercepTest scoring guideline [73]; FGFR3—at least weak but extensive positivity (2) or strong positivity (regardless of extent) (3), as previously described by Tomlinson et al. [74]. Continuous variables were used according to the following formula: H-score = 0 x (% cells with no staining [0]) + 1 x (% cells staining faint, weakly [1+]) + 2 x (% cells staining moderately [2+]) + 3 x (% cells staining strongly [3+]).

### 4.7. Fluorescence In Situ Hybridization (FISH)

*FGFR1* gene amplification using FISH was determined in 18 FFPE tumor samples using in a Zyto*Light* SPEC *FGFR1*/*CEN* 8 Dual Color Probe (containing probes specific for the 8p11 locus and the chromosome 8 centromere (*CEN8*)) (ZytoVision, Bremerhaven, Germany) and Zyto*Light* FISH-Tissue Implementation Kit (ZytoVision) according to the manufacturer’s instructions. Briefly, after pre-treatment, the slides were denatured in the presence of 10 μL of probe for 10 min at 76 °C and hybridized at 37 °C overnight. Sixty tumor cell nuclei were assessed by two independent observers. The criteria of *FGFR1* amplification were as follows: *FGFR1*/*CEN8* ≥ 2.0 or the average number of *FGFR1* signals per cell ≥6 or ≥10% of tumor cells containing ≥ 15 *FGFR1* signals or large clusters [75].

### 4.8. Statistical Analysis

Comparison of the *FGFR1*, *FGFR2*, *FGFR3*, and *FGFR4* gene expression between tumor and tumor-adjacent tissues was performed with the Mann–Whitney U test. Associations between *FGFR1, FGFR2, FGFR3*, and *FGFR4* gene expression and clinicopathological data (Table 1), FGFR1–3 protein expression, and *FGFR1* amplification were analyzed with the Kruskal–Wallis test. To estimate the association between *FGFR* mRNA expression level and clinical endpoint (DFS), univariate Cox proportional hazards model, the Kaplan–Meier method and log-rank test were used. Statistical calculation of correlation and strength of the relationship between mRNA expression levels from RT-PCR and NGS and FGFR protein expression were performed using Spearman’s rank correlation coefficient. mRNA expression was analyzed as a continuous variable and as a categorical variable for Kaplan–Meier analysis (the median value of expression for the entire group was used as a cut-off point). 

A *p*-value < 0.05 was considered significant. All calculations were performed using Statistica software (StatSoft, Tulsa, OK, USA).

## 5. Conclusions

*FGFR1* and *FGFR4* mRNA levels are significantly decreased in Sq-NSCLC tissue compared with tumor-adjacent normal tissue. Furthermore, our study shows that the increased tumor mRNA expression of *FGFR3* is an unfavorable prognostic factor in terms of the risk of recurrence for Sq-NSCLC patients and the increased *FGFR4* mRNA level is correlated with lymph node metastasis occurrence. We also confirm the association of increased *FGFR1* mRNA with protein overexpression but not with *FGFR1* amplification. Moreover, NGS revealed new and well-known *FGFR* variants and fusions. 

*FGFR* mRNA and protein expression analysis in tumor and tumor-adjacent normal tissues, along with the identification of fusions and variants and investigation of amplification status, have increased our knowledge of the molecular background of Sq-NSCLC. Our data also show that the use of different methods increases the detection of FGFR aberrations, which may aid in the selection of patients most likely to respond to treatment with FGFRis.

## Figures and Tables

**Table 1:** Patient characteristics.

Patients n = 40	Frequency	%
**Age (Range 47–80 Years) Median (67 Years)**		
<67 (0)	18	45.00
≥67 (1)	22	55.00
**Gender**		
Male (0)	30	75.00
Female (1)	10	25.00
**Histological type**		
Squamous cell carcinoma	34	85.0
Carcinoma adenosquamous	6	15.0
**Histopathological Grade**		
G1.G2 (0)	25	62.5
G3.G4 (1)	15	37.5
**Clinical stage**		
IA. IB. IIA (0)	21	52.5
IIB. IIIA. IV (1)	18	45.0
Cannot be assessed	1	2.50
**pT-Primary Tumor size**		
≤4 cm (0)	18	45.0
>4 cm (1)	22	55.0
**pN-Regional Lymph Nodes invasion**		
Negative (0)	28	70.0
Positive (1)	11	27.5
Cannot be assessed	1	2.50
**pV-Venous Invasion**		
Negative (0)	24	60.0
Positive (microscopic) (1)	16	40.0
**pL-Lymphatic Invasion**		
Negative (0)	21	52.5
Positive (1)	19	47.5
**pM-Distant metastasis**		
Negative (0)	19	47.5
Positive (1)	1	2.50
Not available	20	50.0
**pR-Residual tumor**		
Negative (0)	35	87.5
Positive (1)	4	10.0
Cannot be assessed	1	2.50
**Actinic keratosis**		
Negative (0)	13	32.5
Positive (1)	25	62.5
Cannot be assessed	2	5.0
**FGFR1 protein expression**		
Negative (0)	14	35.0
Positive (1)	4	10.0
Not assessed	22	55.0
**FGFR2 protein expression**		
Negative (0)	17	42.5
Positive (1)	1	2.5
Not assessed	22	55.0
**FGFR3 protein expression**		
Negative (0)	17	42.5
Positive (1)	1	2.5
Not assessed	22	55.0
***FGFR1* amplification**		
Negative (0)	8	20.0
Positive (1)	7	17.5
Not assessed	25	62.5
**Number of patients at risk of recurrence (DFS)**		
1 year	3	7.5
2 year	4	10.0
3 year	9	12.5
>3 year	5	47.5
No follow up data	19	47.5
**Outcome**		
NED	15	37.5
AWD	6	15.0
DOD	-	-
DOC	-	-
No follow up data	19	47.5

DFS—disease-free survival; NED—no evidence of disease; AWD—alive with disease; DOD—died of disease; DOC—died of other causes; OS—overall survival.

**Table 2:** *FGFR* gene fusions and variants detected in 13 of 40 Sq-NSCLC clinical specimens using RNA-based NGS.

		FGFR RNA Variants							
Patient No	Sample Name	Symbol	HGVSc	HGVSp	Consequence	Clinical Significance	Depth	AO	AF
**1**	M-1	*FGFR2*	c.2419G>A	p.(Glu807Lys)	Missense	Uncertain significance/Benign	796	314	0.39
**2**	M-6	*FGFR2*	c.2211G>T	p.(Met737Ile)	Missense	No information/Pathogenic	700	427	0.61
**3**	M-11	*FGFR2*	c.2398dup	p.(Ser800PhefsTer22)	Frameshift	Uncertain significance/Pathogenic	3136	62	0.01
**4**	M-17	FGFR2	c.2398dup	p.(Ser800PhefsTer22)	frameshift	Uncertain significance/Pathogenic	2624	63	0.02
**5**	M-77	*FGFR2*	c.2398dup	p.(Ser800PhefsTer22)	frameshift	Uncertain significance/Pathogenic	2898	81	0.03
**6**	M-88	*FGFR1*	c.899T>C	p.(Ile300Thr)	missense	Uncertain significance/Uncertain significance	278	184	0.66
**7**	M-115	*FGFR3*	c.746C>G	p.(Ser249Cys) (S249C)	missense	Pathogenic/Pathogenic	251	124	0.49
**8**	M-119	*FGFR2*	c.870G>T	p.(Trp290Cys) (W290C)	missense	Pathogenic/Pathogenic	304	24	0.08
**9**	M-123	*FGFR2*	c.2398dup	p.(Ser800PhefsTer22)	frameshift	Uncertain significance/Pathogenic	3016	70	0.02
**10**	M-135	*FGFR2*	c.2398dup	p.(Ser800PhefsTer22)	frameshift	Uncertain significance/Pathogenic	1189	30	0.02
	M-135P	*FGFR2*	c.2398dup	p.(Ser800PhefsTer22)	frameshift	Uncertain significance/Pathogenic	643	15	0.02
**11**	M-138	*FGFR2*	c.2398dup	p.(Ser800PhefsTer22)	frameshift	Uncertain significance/Pathogenic	1633	40	0.02
	M-138P	nd							
		**FGFR RNA Fusions**							
**Patient no**	**Sample name**	**Symbol**	**SS**	**Reads**	**%Reads**	**Breakpoint**			
**12**	M-20	FGFR3::TACC3	17	35	5.49	chr4:1808661.chr4:1741429			
	M-20P	nd							
**13**	M-61	TACC1::FGFR1	17	25	4.66	chr8:38646337.chr8:38315052			
	M-61P	nd							

M-X—tumors; M-XP—tumor-adjacent normal tissues; nd—no fusion and/or variant detected; SS—number of supportive reads with unique start sites; Reads—total number of supportive reads; %Reads—percent of reads at breakpoint supporting fusion; Breakpoint—the breakpoints associated with the event, in hg19 coordinates; Depth—the total high quality unique molecule depth covering the variant; AO—total number of reads that support the alternate allele; AF—the allele fraction (AF) of the reads that support the alternative allele. Clinical Significance—clinical interpretation of sequence variants based on ClinVar* and Varsome** databases; Reference transcripts: *FGFR1*: NM_023110.3, NP_075598.2; *FGFR2*: NM_022970.3, NP_075259.4; *FGFR3*: NM_000142.4, NP_000133.1; *TACC1*: NM_006283.2; *TACC3*: NM_006342.2

## Data Availability

Not applicable.

## Supplementary Materials

The supporting information can be downloaded at: https://www.mdpi.com/article/10.3390/ijms231810506/s1.

## References

[1] Ferlay J., Colombet M., Soerjomataram I., Parkin D.M., Piñeros M., Znaor A., Bray F. (2021). Cancer Statistics for the Year 2020: An Overview. Int. J. Cancer.

[2] Reck M., Remon J., Hellmann M.D. (2022). First-Line Immunotherapy for Non-Small-Cell Lung Cancer. J. Clin. Oncol..

[3] Reck M., Rodríguez-Abreu D., Robinson A.G., Hui R., Csőszi T., Fülöp A., Gottfried M., Peled N., Tafreshi A., Cuffe S. (2016). Pembrolizumab versus Chemotherapy for PD-L1–Positive Non–Small-Cell Lung Cancer. N. Engl. J. Med..

[4] (2012). Cancer Genome Atlas Research Network Comprehensive Genomic Characterization of Squamous Cell Lung Cancers. Nature.

[5] Babina I.S., Turner N.C. (2017). Advances and Challenges in Targeting FGFR Signalling in Cancer. Nat. Rev. Cancer.

[6] Hashemi-Sadraei N., Hanna N. (2017). Targeting FGFR in Squamous Cell Carcinoma of the Lung. Target. Oncol..

[7] Moes-Sosnowska J., Chorostowska-Wynimko J. (2022). Fibroblast Growth Factor Receptor 1-4 Genetic Aberrations as Clinically Relevant Biomarkers in Squamous Cell Lung Cancer. Front. Oncol..

[8] Nogova L., Sequist L.V., Perez Garcia J.M., Andre F., Delord J.-P., Hidalgo M., Schellens J.H.M., Cassier P.A., Camidge D.R., Schuler M. (2017). Evaluation of BGJ398, a Fibroblast Growth Factor Receptor 1-3 Kinase Inhibitor, in Patients with Advanced Solid Tumors Harboring Genetic Alterations in Fibroblast Growth Factor Receptors: Results of a Global Phase I, Dose-Escalation and Dose-Expansion Study. J. Clin. Oncol..

[9] Aggarwal C., Redman M.W., Lara P.N., Borghaei H., Hoffman P., Bradley J.D., Newman A.J., Feldman M.J., Minichiello K., Miao J. (2019). SWOG S1400D (NCT02965378), a Phase II Study of the Fibroblast Growth Factor Receptor Inhibitor AZD4547 in Previously Treated Patients with Fibroblast Growth Factor Pathway-Activated Stage IV Squamous Cell Lung Cancer (Lung-MAP Substudy). J. Thorac. Oncol..

[10] Paik P.K., Shen R., Berger M.F., Ferry D., Soria J.-C., Mathewson A., Rooney C., Smith N.R., Cullberg M., Kilgour E. (2017). A Phase Ib Open-Label Multicenter Study of AZD4547 in Patients with Advanced Squamous Cell Lung Cancers. Clin. Cancer Res..

[11] Andre F., Ranson M., Dean E., Varga A., van der Noll R., Stockman P.K., Ghiorghiu D., Kilgour E., Smith P.D., Macpherson M. (2013). Abstract LB-145: Results of a Phase I Study of AZD4547, an Inhibitor of Fibroblast Growth Factor Receptor (FGFR), in Patients with Advanced Solid Tumors. Cancer Res..

[12] Rooney C., Geh C., Williams V., Heuckmann J.M., Menon R., Schneider P., Al-Kadhimi K., Dymond M., Smith N.R., Baker D. (2016). Characterization of FGFR1 Locus in SqNSCLC Reveals a Broad and Heterogeneous Amplicon. PLoS ONE.

[13] Ren M., Hong M., Liu G., Wang H., Patel V., Biddinger P., Silva J., Cowell J., Hao Z. (2013). Novel FGFR Inhibitor Ponatinib Suppresses the Growth of Non-Small Cell Lung Cancer Cells Overexpressing FGFR1. Oncol. Rep..

[14] Wynes M.W., Hinz T.K., Gao D., Martini M., Marek L.A., Ware K.E., Edwards M.G., Böhm D., Perner S., Helfrich B.A. (2014). FGFR1 MRNA and Protein Expression, Not Gene Copy Number, Predict FGFR TKI Sensitivity across All Lung Cancer Histologies. Clin. Cancer Res..

[15] Grünewald S., Politz O., Bender S., Héroult M., Lustig K., Thuss U., Kneip C., Kopitz C., Zopf D., Collin M.-P. (2019). Rogaratinib: A Potent and Selective Pan-FGFR Inhibitor with Broad Antitumor Activity in FGFR-Overexpressing Preclinical Cancer Models. Int. J. Cancer.

[16] Addeo A., Joerger M., Rothschild S., Eboulet E.I., Godar G., Waibel-Pachinger C., Haefliger S., Mark M.T., Fernandez E., Mach N. (2021). Fibroblast Growth Factor Receptor (FGFR) Inhibitor Rogaratinib in Patients with Advanced Pretreated Squamous-Cell Non-Small Cell Lung Cancer over-Expressing FGFR MRNA: The SAKK 19/18 Phase II Study. J. Clin. Oncol..

[17] Ng T.L., Yu H., Smith D.E., Boyle T.A., York E.R., Leedy S., Gao D., Aisner D.L., Van Bokhoven A., Heasley L.E. (2019). Preselection of Lung Cancer Cases Using FGFR1 MRNA and Gene Copy Number for Treatment with Ponatinib. Clin. Lung Cancer.

[18] Ornitz D.M., Itoh N. (2015). The Fibroblast Growth Factor Signaling Pathway. Wiley Interdiscip. Rev. Dev. Biol..

[19] Bogatyrova O., Mattsson J.S.M., Ross E.M., Sanderson M.P., Backman M., Botling J., Brunnström H., Kurppa P., La Fleur L., Strell C. (2021). FGFR1 Overexpression in Non-Small Cell Lung Cancer Is Mediated by Genetic and Epigenetic Mechanisms and Is a Determinant of FGFR1 Inhibitor Response. Eur. J. Cancer.

[20] Gresner P., Gromadzinska J., Wasowicz W. (2009). Reference Genes for Gene Expression Studies on Non-Small Cell Lung Cancer. Acta Biochim. Pol..

[21] Smith T.A.D., AbdelKarem O.A., Irlam-Jones J.J., Lane B., Valentine H., Bibby B.A.S., Denley H., Choudhury A., West C.M.L. (2020). Selection of Endogenous Control Genes for Normalising Gene Expression Data Derived from Formalin-Fixed Paraffin-Embedded Tumour Tissue. Sci. Rep..

[22] Moes-Sosnowska J., Szczepulska-Wojcik E., Rozy A., Rudzinski S., Langfort R., Rudzinski P., Orlowski T., Chorostowska-Wynimko J. (2019). FGFR1–4 and MET Expression Analysis and Evaluation of Reliable Reference Genes in Sq-NSCLC. Eur. Respir. J..

[23] Huang H.-P., Feng H., Qiao H.-B., Ren Z.-X., Zhu G.-D. (2015). The Prognostic Significance of Fibroblast Growth Factor Receptor 4 in Non-Small-Cell Lung Cancer. Onco. Targets. Ther..

[24] Justet A., Ghanem M., Jaillet M., Hachem M., Boghanim T., Vadel A., Mailleux A., Crestani B. (2020). FGFR4 Has pro Fibrotic Properties in Idiopathic Pulmonary Fibrosis. Eur. Respir. J..

[25] Quintanal-Villalonga Á., Ferrer I., Guruceaga E., Cirauqui C., Marrugal Á., Ojeda L., García S., Zugazagoitia J., Muñoz-Galván S., Lopez-Rios F. (2020). FGFR1 and FGFR4 Oncogenicity Depends on N-Cadherin and Their Co-Expression May Predict FGFR-Targeted Therapy Efficacy. EBioMedicine.

[26] Wang L., Ren Z., Yu B., Tang J. (2021). Development of Nomogram Based on Immune-Related Gene FGFR4 for Advanced Non-Small Cell Lung Cancer Patients with Sensitivity to Immune Checkpoint Inhibitors. J. Transl. Med..

[27] Wei W., Cao S., Liu J., Wang Y., Song Q., Leha A., Sun S., Zhang X., Liang X., Jiang Y. (2020). Fibroblast Growth Factor Receptor 4 as a Prognostic Indicator in Triple-Negative Breast Cancer. Transl. Cancer Res..

[28] Inokuchi M., Murase H., Otsuki S., Kawano T., Kojima K. (2017). Different Clinical Significance of FGFR1–4 Expression between Diffuse-Type and Intestinal-Type Gastric Cancer. World J. Surg. Oncol..

[29] Murase H., Inokuchi M., Takagi Y., Kato K., Kojima K., Sugihara K. (2014). Prognostic Significance of the Co-Overexpression of Fibroblast Growth Factor Receptors 1, 2 and 4 in Gastric Cancer. Mol. Clin. Oncol..

[30] Jaakkola S., Salmikangas P., Nylund S., Partanen J., Armstrong E., Pyrhönen S., Lehtovirta P., Nevanlinna H. (1993). Amplification of Fgfr4 Gene in Human Breast and Gynecological Cancers. Int. J. Cancer.

[31] Quintanal-Villalonga Á., Carranza-Carranza A., Meléndez R., Ferrer I., Molina-Pinelo S., Paz-Ares L. (2017). Prognostic Role of the FGFR4-388Arg Variant in Lung Squamous-Cell Carcinoma Patients with Lymph Node Involvement. Clin. Lung Cancer.

[32] Wang J., Yu W., Cai Y., Ren C., Ittmann M.M. (2008). Altered Fibroblast Growth Factor Receptor 4 Stability Promotes Prostate Cancer Progression. Neoplasia.

[33] Nannapaneni S., Griffith C.C., Magliocca K.R., Chen W., Lyu X., Chen Z., Wang D., Wang X., Shin D.M., Chen Z.G. (2021). Co-Expression of Fibroblast Growth Factor Receptor 3 with Mutant P53, and Its Association with Worse Outcome in Oropharyngeal Squamous Cell Carcinoma. PLoS ONE.

[34] Sikic D., Taubert H., Breyer J., Eckstein M., Weyerer V., Keck B., Kubon J., Otto W., Worst T.S., Kriegmair M.C. (2021). The Prognostic Value of FGFR3 Expression in Patients with T1 Non-Muscle Invasive Bladder Cancer. Cancer Manag. Res..

[35] Jing P., Zhao N., Xie N., Ye M., Zhang Y., Zhang Z., Li M., Lai X., Zhang J., Gu Z. (2018). MiR-24-3p/FGFR3 Signaling as a Novel Axis Is Involved in Epithelial-Mesenchymal Transition and Regulates Lung Adenocarcinoma Progression. J. Immunol. Res..

[36] Starska K., Forma E., Lewy-Trenda I., Stasikowska-Kanicka O., Skóra M., Bryś M. (2018). Fibroblast Growth Factor Receptor 1 and 3 Expression Is Associated with Regulatory PI3K/AKT Kinase Activity, as Well as Invasion and Prognosis, in Human Laryngeal Cancer. Cell. Oncol..

[37] Kang H.W., Kim Y.-H., Jeong P., Park C., Kim W.T., Ryu D.H., Cha E.-J., Ha Y.-S., Kim T.-H., Kwon T.G. (2017). Expression Levels of FGFR3 as a Prognostic Marker for the Progression of Primary PT1 Bladder Cancer and Its Association with Mutation Status. Oncol. Lett..

[38] Weiss J., Sos M.L., Seidel D., Peifer M., Zander T., Heuckmann J.M., Ullrich R.T., Menon R., Maier S., Soltermann A. (2010). Frequent and Focal FGFR1 Amplification Associates with Therapeutically Tractable FGFR1 Dependency in Squamous Cell Lung Cancer. Sci. Transl. Med..

[39] Gil F., Miranda-Filho A., Uribe-Perez C., Arias-Ortiz N.E., Yépez-Chamorro M.C., Bravo L.M., de Vries E. (2022). Impact of the Management and Proportion of Lost to Follow-up Cases on Cancer Survival Estimates for Small Population-Based Cancer Registries. J. Cancer Epidemiol..

[40] Kang D., Jung J., Park S., Cho B.-S., Kim H.-J., Kim Y., Lee J.-M., Kim H.S., Ahn A., Kim M. (2022). Genetic Characteristics According to Subgroup of Acute Myeloid Leukemia with Myelodysplasia-Related Changes. J. Clin. Med..

[41] Desmeules P., Boudreau D.K., Bastien N., Boulanger M.-C., Bossé Y., Joubert P., Couture C. (2022). Performance of an RNA-Based next-Generation Sequencing Assay for Combined Detection of Clinically Actionable Fusions and Hotspot Mutations in NSCLC. JTO Clin. Res. Rep..

[42] Guseva N.V., Jaber O., Tanas M.R., Stence A.A., Sompallae R., Schade J., Fillman A.N., Miller B.J., Bossler A.D., Ma D. (2017). Anchored Multiplex PCR for Targeted Next-Generation Sequencing Reveals Recurrent and Novel USP6 Fusions and Upregulation of USP6 Expression in Aneurysmal Bone Cyst. Genes Chromosomes Cancer.

[43] Sands J.M., Nguyen T., Shivdasani P., Sacher A.G., Cheng M.L., Alden R.S., Jänne P.A., Kuo F.C., Oxnard G.R., Sholl L.M. (2020). Next-Generation Sequencing Informs Diagnosis and Identifies Unexpected Therapeutic Targets in Lung Squamous Cell Carcinomas. Lung Cancer.

[44] Theelen W.S., Mittempergher L., Willems S.M., Bosma A.J., Peters D.D., van der Noort V., Japenga E.J., Peeters T., Koole K., Šuštić T. (2016). FGFR1, 2 and 3 Protein Overexpression and Molecular Aberrations of FGFR3 in Early Stage Non-Small Cell Lung Cancer: FGFR Expression in Early Stage Non-Small Lung Cancer. J. Pathol. Clin. Res..

[45] Hibi M., Kaneda H., Tanizaki J., Sakai K., Togashi Y., Terashima M., De Velasco M.A., Fujita Y., Banno E., Nakamura Y. (2016). FGFR Gene Alterations in Lung Squamous Cell Carcinoma Are Potential Targets for the Multikinase Inhibitor Nintedanib. Cancer Sci..

[46] Lim S.H., Sun J.-M., Choi Y.-L., Kim H.R., Ahn S., Lee J.Y., Lee S.-H., Ahn J.S., Park K., Kim J.H. (2016). Efficacy and Safety of Dovitinib in Pretreated Patients with Advanced Squamous Non-Small Cell Lung Cancer with FGFR1 Amplification: A Single-Arm, Phase 2 Study: Dovitinib InFGFR1-Amplified SCC. Cancer.

[47] Wu Y.-M., Su F., Kalyana-Sundaram S., Khazanov N., Ateeq B., Cao X., Lonigro R.J., Vats P., Wang R., Lin S.-F. (2013). Identification of Targetable FGFR Gene Fusions in Diverse Cancers. Cancer Discov..

[48] Stransky N., Cerami E., Schalm S., Kim J.L., Lengauer C. (2014). The Landscape of Kinase Fusions in Cancer. Nat. Commun..

[49] Wang R., Wang L., Li Y., Hu H., Shen L., Shen X., Pan Y., Ye T., Zhang Y., Luo X. (2014). FGFR1/3 Tyrosine Kinase Fusions Define a Unique Molecular Subtype of Non-Small Cell Lung Cancer. Clin. Cancer Res..

[50] Qin A., Johnson A., Ross J.S., Miller V.A., Ali S.M., Schrock A.B., Gadgeel S.M. (2019). Detection of Known and Novel FGFR Fusions in Non-Small Cell Lung Cancer by Comprehensive Genomic Profiling. J. Thorac. Oncol..

[51] Kim Y., Hammerman P.S., Kim J., Yoon J.-A., Lee Y., Sun J.-M., Wilkerson M.D., Pedamallu C.S., Cibulskis K., Yoo Y.K. (2014). Integrative and Comparative Genomic Analysis of Lung Squamous Cell Carcinomas in East Asian Patients. J. Clin. Oncol..

[52] Zhou Z., Liu Z., Ou Q., Wu X., Wang X., Shao Y., Liu H., Yang Y. (2021). Targeting FGFR in Non-Small Cell Lung Cancer: Implications from the Landscape of Clinically Actionable Aberrations of FGFR Kinases. Cancer Biol. Med..

[53] Gozgit J.M., Wong M.J., Moran L., Wardwell S., Mohemmad Q.K., Narasimhan N.I., Shakespeare W.C., Wang F., Clackson T., Rivera V.M. (2012). Ponatinib (AP24534), a Multitargeted Pan-FGFR Inhibitor with Activity in Multiple FGFR-Amplified or Mutated Cancer Models. Mol. Cancer Ther..

[54] Schittenhelm J., Ziegler L., Sperveslage J., Mittelbronn M., Capper D., Burghardt I., Poso A., Biskup S., Skardelly M., Tabatabai G. (2021). FGFR3 Overexpression Is a Useful Detection Tool for FGFR3 Fusions and Sequence Variations in Glioma. Neurooncol. Pract..

[55] Parker B.C., Annala M.J., Cogdell D.E., Granberg K.J., Sun Y., Ji P., Li X., Gumin J., Zheng H., Hu L. (2013). The Tumorigenic FGFR3-TACC3 Gene Fusion Escapes MiR-99a Regulation in Glioblastoma. J. Clin. Investig..

[56] Helsten T., Elkin S., Arthur E., Tomson B.N., Carter J., Kurzrock R. (2016). The FGFR Landscape in Cancer: Analysis of 4,853 Tumors by next-Generation Sequencing. Clin. Cancer Res..

[57] Liao R.G., Jung J., Tchaicha J., Wilkerson M.D., Sivachenko A., Beauchamp E.M., Liu Q., Pugh T.J., Pedamallu C.S., Hayes D.N. (2013). Inhibitor-Sensitive FGFR2 and FGFR3 Mutations in Lung Squamous Cell Carcinoma. Cancer Res..

[58] Dutt A., Salvesen H.B., Chen T.-H., Ramos A.H., Onofrio R.C., Hatton C., Nicoletti R., Winckler W., Grewal R., Hanna M. (2008). Drug-Sensitive FGFR2 Mutations in Endometrial Carcinoma. Proc. Natl. Acad. Sci. USA.

[59] Majewski I.J., Mittempergher L., Davidson N.M., Bosma A., Willems S.M., Horlings H.M., de Rink I., Greger L., Hooijer G.K.J., Peters D. (2013). Identification of Recurrent FGFR3 Fusion Genes in Lung Cancer through Kinome-Centred RNA Sequencing: Kinase Fusion Genes in NSCLC. J. Pathol..

[60] Flockerzi F.A., Roggia C., Langer F., Holleczek B., Bohle R.M. (2018). FGFR1 Gene Amplification in Squamous Cell Carcinomas of the Lung: A Potential Favorable Prognostic Marker for Women and for Patients with Advanced Cancer. Virchows Arch..

[61] Nakamura I.T., Kohsaka S., Ikegami M., Ikeuchi H., Ueno T., Li K., Beyett T.S., Koyama T., Shimizu T., Yamamoto N. (2021). Comprehensive Functional Evaluation of Variants of Fibroblast Growth Factor Receptor Genes in Cancer. NPJ Precis. Oncol..

[62] Wilkie A.O.M., Bochukova E.G., Hansen R.M.S., Taylor I.B., Rannan-Eliya S.V., Byren J.C., Wall S.A., Ramos L., Venâncio M., Hurst J.A. (2006). Clinical Dividends from the Molecular Genetic Diagnosis of Craniosynostosis. Am. J. Med. Genet. A.

[63] Kress W., Petersen B., Collmann H., Grimm T. (2000). An Unusual FGFR1 Mutation (Fibroblast Growth Factor Receptor 1 Mutation) in a Girl with Non-Syndromic Trigonocephaly. Cytogenet. Cell Genet..

[64] Richards S., Aziz N., Bale S., Bick D., Das S., Gastier-Foster J., Grody W.W., Hegde M., Lyon E., Spector E. (2015). Standards and Guidelines for the Interpretation of Sequence Variants: A Joint Consensus Recommendation of the American College of Medical Genetics and Genomics and the Association for Molecular Pathology. Genet. Med..

[65] Joerger M., Cho B.C., Mach N., Caballero C., Soo R.A., Wirth M., Sayehli C., Navarro A., Keam B., Piciu A.-M. (2019). Early Clinical Experience with the Pan-FGFR Inhibitor Rogaratinib in Patients with Non-Small Cell Lung Cancer Selected Based on FGFR MRNA Expression Levels. J. Clin. Oncol..

[66] De Luca A., Esposito Abate R., Rachiglio A.M., Maiello M.R., Esposito C., Schettino C., Izzo F., Nasti G., Normanno N. (2020). FGFR Fusions in Cancer: From Diagnostic Approaches to Therapeutic Intervention. Int. J. Mol. Sci..

[67] Bahleda R., Italiano A., Hierro C., Mita A., Cervantes A., Chan N., Awad M., Calvo E., Moreno V., Govindan R. (2019). Multicenter Phase I Study of Erdafitinib (JNJ-42756493), Oral Pan-Fibroblast Growth Factor Receptor Inhibitor, in Patients with Advanced or Refractory Solid Tumors. Clin. Cancer Res..

[68] Travis W.D., Brambilla E., Burke A.P., Marx A., Nicholson A.G. (2015). Introduction to the 2015 World Health Organization Classification of Tumors of the Lung, Pleura, Thymus, and Heart. J. Thorac. Oncol..

[69] Brierley J.D., Gospodarowicz M.K., Wittekind C. (2016). TNM Classification of Malignant Tumours.

[70] Skupinska M.M., Obtulowicz T., Moes-Sosnowska J., Rozy A., Szczepulska E., Langfort R., Wynimko J.C., Stanczak A., Pieczykolan J., Wieczorek M. (2020). 1409P Comparing Different Methods of FGFR1 Aberrations Analysis in Squamous Cell Lung Cancer (SqCLC) Targeted Therapy. Ann. Oncol..

[71] Skupinska M.M., Jesiotr M., Chrom P., Mroz A., Cierniak S., Winiarek M., Wyrwicz L.S., Pieczykolan J., Wieczorek M., Stanczak A. (2018). The Role of FGFR2 Amplification and Expression in Patients with Advanced or Metastatic Gastric Cancer Receiving Fluoropyrimidine-Based Chemotherapy. Ann. Oncol..

[72] Sosnowski R., Popiel D., Gapska P., Skupińska M., Stajno P., Sobieszek-Prochorec M., Ligaj M., Demkow T., Stańczak A., Wieczorek M. (2021). 712P Analysis of Fibroblast Growth Factor Receptor 3 Aberrations in Bladder Cancer, for Enabling Personalized and Effective Therapy Based on FGFR Inhibitor. Ann. Oncol..

[73] Dowsett M., Bartlett J., Ellis I.O., Salter J., Hills M., Mallon E., Watters A.D., Cooke T., Paish C., Wencyk P.M. (2003). Correlation between Immunohistochemistry (HercepTest) and Fluorescence in Situ Hybridization (FISH) for HER-2 in 426 Breast Carcinomas from 37 Centres. J. Pathol..

[74] Tomlinson D.C., Baldo O., Harnden P., Knowles M.A. (2007). FGFR3 Protein Expression and Its Relationship to Mutation Status and Prognostic Variables in Bladder Cancer. J. Pathol..

[75] Schultheis A.M., Bos M., Schmitz K., Wilsberg L., Binot E., Wolf J., Büttner R., Schildhaus H.-U. (2014). Fibroblast Growth Factor Receptor 1 (FGFR1) Amplification Is a Potential Therapeutic Target in Small-Cell Lung Cancer. Mod. Pathol..

