# Supplementary material for: FGFR1–4 RNA-Based Gene Alteration and Expression Analysis in Squamous Non-Small Cell Lung Cancer
Source: Int J Mol Sci. 2022 Sep 10;23(18):10506. doi: 10.3390/ijms231810506 (PMC9505002; doi:10.3390/ijms231810506)
Supplement: Supplementary file 1 [file ijms-23-10506-s001.zip › ijms-1864175-supplementary.pdf]

Supplementary Data for

FGFR1–4 RNA-Based Gene Alteration and Expression Analysis in Squamous Non-Small Cell Lung Cancer

Joanna Moes-Sosnowska[1], Monika Skupinska [2], Urszula Lechowicz [1] , Ewa Szczepulska-Wojcik [3], Paulina Skronska [1], Adriana Rozy [1], Aneta Stepniewska [1], Renata Langfort [3], Piotr Rudzinski [4], Tadeusz Orlowski [4], Delfina Popiel [2], Aleksandra Stanczak [5], Maciej Wieczorek [2, 5], Joanna Chorostowska-Wynimko [1*]

1 Department of Genetics and Clinical Immunology, National Institute of Tuberculosis and Lung Diseases, 01-138 Warsaw, Poland;
2 Preclinical Development Department, Celon Pharma S.A, Research & Development Centre, 05-152 Kazun Nowy, Poland;
3 Department of Pathology, National Institute of Tuberculosis and Lung Diseases, 01-138 Warsaw, Poland;
4 Department of Surgery, National Institute of Tuberculosis and Lung Diseases, 01-138 Warsaw, Poland;
5 Clinical Development Department, Celon Pharma S.A., Research & Development Centre, 05-152 Kazun Nowy, Poland;
* Correspondence: j.chorostowska@gmail.com or j.chorostowska@igichp.edu.pl

This PDF file includes:

Supplementary Tables S1 to S2
Supplementary Figures S1 to S5

Moes-Sosnowska, J.; Skupinska, M.; Lechowicz, U.; Szczepulska-Wojcik, E.; Skronska, P.; Rozy, A.; Stepniewska, A.; Langfort, R.; Rudzinski, P.; Orlowski, T.; et al. FGFR1–4 RNA-Based Gene Alteration and Expression Analysis in Squamous Non-Small Cell Lung Cancer. *Int. J. Mol. Sci.* 2022, *23*

Supplementary Tables

Table S1. Fusions and oncogenic isoforms revealed with use of the Archer FusionPlex Lung gene panel in the commercial control sample Seraseq (QC 242.50).

	Genes	SS	Reads	%Reads	Strong	Breakpoint	Category
1	CD74::ROS1	18	30	41.1	True	chr5:149784243,chr6:117645578	Fusion
2	EGFR	124	373	93.02	True	chr7:55087058,chr7:55223523	Oncogenic Isoform
3	EGFR::SEPT14	91	122	92.42	True	chr7:55268106,chr7:55863785	Fusion
4	EML4::ALK	65	316	96.05	True	chr2:42522656,chr2:29446394	Fusion
5	ETV6::NTRK3	55	143	100.0	True	chr12:12022903,chr15:88483984	Fusion
6	FGFR3::BAIAP2L1	45	234	50.11	True	chr4:1808661,chr7:97991744	Fusion
7	FGFR3::TACC3	57	255	51.41	True	chr4:1808661,chr4:1741429	Fusion
8	KIF5B::RET	80	266	97.79	True	chr10:32306071,chr10:43609928	Fusion
9	LMNA::NTRK1	62	175	42.89	True	chr1:156100564,chr1:156844698	Fusion
10	MET	17	25	69.44	True	chr7:116411708,chr7:116414935	Oncogenic Isoform
11	NCOA4::RET	69	234	63.93	True	chr10:51582939,chr10:43612032	Fusion
12	SLC34A2::ROS1	15	32	40.0	True	chr4:25665952,chr6:117645578	Fusion
13	SLC45A3::BRAF	51	280	3.67	True	chr1:205649522,chr7:140494267	Fusion
14	TPM3::NTRK1	78	305	96.52	True	chr1:154142876,chr1:156844363	Fusion

SS—number of supportive reads with unique start sites; Reads—total number of supportive reads; %Reads—percent of reads at breakpoint supporting fusion; Breakpoint—the breakpoints associated with the event, in hg19 coordinates;

Table S2. Sample reads statistics summary (Archer FusionPlex lung NGS panel).

	Read Statistics			RNA Statistics		
Sample	Total Fragments	All Fragments	Unique Fragments / (%)	All RNA Reads (# / %)	Molecular Bins	QC
M-1	442458	358947	295337 / 82.3	285420 / 80.9	237167	426.00
M-3	818403	685516	303863 / 44.3	553148 / 82.8	257993	503.88
M-6	1564753	1189940	480504 / 40.4	961445 / 81.9	416735	585.25
M-11	448852	365124	208142 / 57.0	268863 / 75.9	158315	370.12
M-17	1382865	1103500	457953 / 41.5	892296 / 82.4	382548	501.62
M-19	1045908	893791	273189 / 30.6	776767 / 88.4	241792	542.00
M-20	1140463	919577	295755 / 32.2	738436 / 81.8	251683	545.50
M-28	582092	491342	288658 / 58.7	417189 / 86.2	250578	484.25
M-33	838437	688660	315924 / 45.9	510414 / 76.3	246180	535.75
M-35	765340	607019	391324 / 64.5	475675 / 80.5	311595	422.62
M-37	386620	323577	219068 / 67.7	261060 / 82.3	180653	449.62
M-51	1134709	906409	471468 / 52.0	772485 / 86.7	413239	493.12
M-61	878679	725365	270845 / 37.3	601831 / 84.2	235048	494.75
M-67	1248332	975409	406313 / 41.7	774395 / 81.3	333586	540.62

M-70	1089699	855179	314621 / 36.8	686653 / 82.5	265252	529.75
M-77	1737707	1298013	319942 / 24.6	1042421 / 81.5	272117	563.62
M-83	1379884	1156964	507774 / 43.9	946898 / 83.4	429997	557.5
M-88	578896	487094	304241 / 62.5	407034 / 84.8	261356	469.0
M-89	1442977	1169658	146090 / 12.5	834129 / 76.9	125910	390.0
M-105	657090	500899	369633 / 73.8	416322 / 85.3	311313	440.62
M-106	1531962	1188924	575429 / 48.4	927132 / 79.3	466665	460.38
M-108	1640788	1297094	574905 / 44.3	1122865 / 87.7	495717	535.50
M-112	1157123	904041	457529 / 50.6	737722 / 83.2	385149	471.12
M-113	1608493	1353020	713456 / 52.7	1089826 / 81.9	585501	582.88
M-115	669330	499009	278553 / 55.8	386227 / 79.4	222485	460.62
M-119	1167205	928227	403116 / 43.4	754229 / 82.4	343239	524.50
M-123	1551304	1253157	487248 / 38.9	1038901 / 84.3	422418	607.38
M-129	1399912	1104962	458790 / 41.5	931476 / 85.7	399916	558.50
M-134	1126443	868952	533402 / 61.4	732420 / 85.7	456270	521.62
M-135	735821	561047	178503 / 31.8	477560 / 86.8	154264	435.12
M-138	1124711	900483	350051 / 38.9	740566 / 84.1	303917	421.88
M-142	1168796	936669	545792 / 58.3	776320 / 84.3	453395	516.88
M-146	1975114	1636254	709671 / 43.4	1309238 / 82.3	595277	589.88
M-155	1198981	933605	431032 / 46.2	742163 / 80.9	359546	547.00
M-176	1382865	1103500	457953 / 41.5	892296 / 82.4	382548	593.88
M-180	1766592	1403860	714452 / 50.9	1128166 / 81.5	582685	525.38
M-183	1139587	900796	357327 / 39.7	755451 / 85.1	312418	580.00
M-186	1791864	1366694	496316 / 36.3	1139835 / 84.9	420990	570.50
M-187	1277150	1005365	331151 / 32.9	777421 / 79.4	266981	541.75
M-189	1568574	1225003	568216 / 46.4	955858 / 79.4	461389	524.62
M-20P	1643433	1306916	265691 / 20.3	1053900 / 82.4	219796	491.00
M-33P	1101458	844861	285215 / 33.8	658295 / 80.1	234084	467.00
M-61P	1142129	897597	209639 / 23.4	719612 / 82.2	177342	493.12
M-135P	638460	491283	193600 / 39.4	395420 / 82.8	161865	447.75
M-138P	690217	508685	120568 / 23.7	390375 / 79.6	96430	387.38
Seraseq	1314536	960282	107686 / 11.2	678839 / 73.5	71736	242.50
HORIZON	3500000	2762989	284911 / 10.3	1983390 / 73.1	187256	285.50

Total Fragments—Total number of read (pairs) that were present in the original FASTQ file; All Fragments—indicates the non-deduplicated raw read(s) or read pairs; Unique Fragments—indicates the de-duplicated reads, based on either the alignment or the molecular barcode; All RNA Reads (# / %) Total and percentage of reads that likely come from an RNA source; reads that span exon-exon splice junctions. indicates the non-deduplicated raw read(s) or read pairs; Molecular Bins—Unique fragments/molecules from the original sample, as determined by use of molecular barcodes (MBCs) and deduplication. Molecular bins are defined as reads having the same random molecular barcode in the ligated adapter. This also requires the reads to have all the same start sites. The same MBC sequence could be seen in different start site locations. QC—Average Unique RNA Start Sites per Control GSP2

Moes-Sosnowska, J.; Skupinska, M.; Lechowicz, U.; Szczepulska-Wojcik, E.; Skronska, P.; Rozy, A.; Stepniewska, A.; Langfort, R.; Rudzinski, P.; Orlowski, T.; et al. FGFR1–4 RNA-Based Gene Alteration and Expression Analysis in Squamous Non-Small Cell Lung Cancer. *Int. J. Mol. Sci.* 2022, *23*

Supplementary Figures

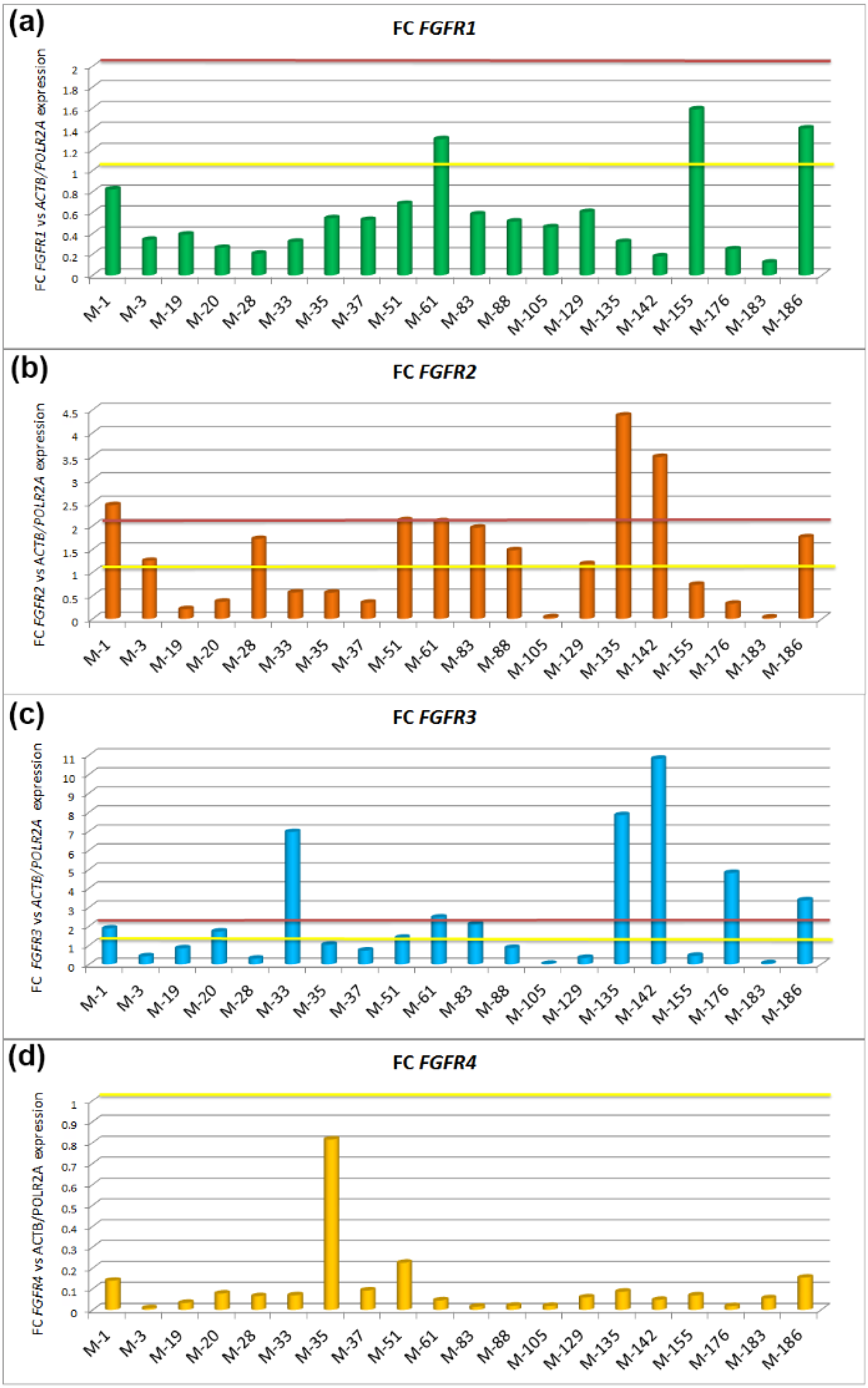

Figure S1. Fold change of RT-PCR *FGFR1–4* gene expression (**a**–**d**) in Sq-NSCLC vs. corresponding tumor-adjacent normal tissues (n = 20). The bars on the graphs over the baseline (fold change > 1) indicate the increase in gene expression compared to adjacent normal tissue from the same patient, while the bars under the baseline (fold change < 1) indicate a decrease in gene expression.

Moes-Sosnowska, J.; Skupinska, M.; Lechowicz, U.; Szczepulska-Wojcik, E.; Skronska, P.; Rozy, A.; Stepniewska, A.; Langfort, R.; Rudzinski, P.; Orlowski, T.; et al. FGFR1–4 RNA-Based Gene Alteration and Expression Analysis in Squamous Non-Small Cell Lung Cancer. *Int. J. Mol. Sci.* 2022, *23*

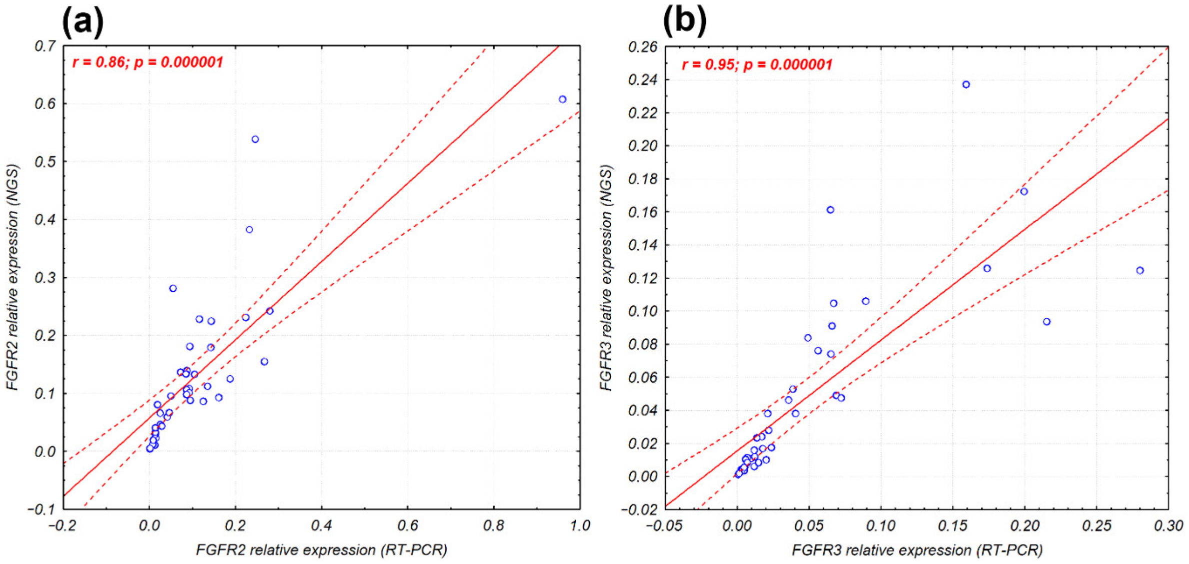

Figure S2. Correlation scatter plot of the (**a**) *FGFR2* and (**b**) *FGFR3* gene expression assessed by RT-PCR and NGS (Archer Lung FusionPlex) in 40 Sq-NSCLC tumor samples.

Moes-Sosnowska, J.; Skupinska, M.; Lechowicz, U.; Szczepulska-Wojcik, E.; Skronska, P.; Rozy, A.; Stepniewska, A.; Langfort, R.; Rudzinski, P.; Orlowski, T.; et al. FGFR1–4 RNA-Based Gene Alteration and Expression Analysis in Squamous Non-Small Cell Lung Cancer. *Int. J. Mol. Sci.* 2022, *23*

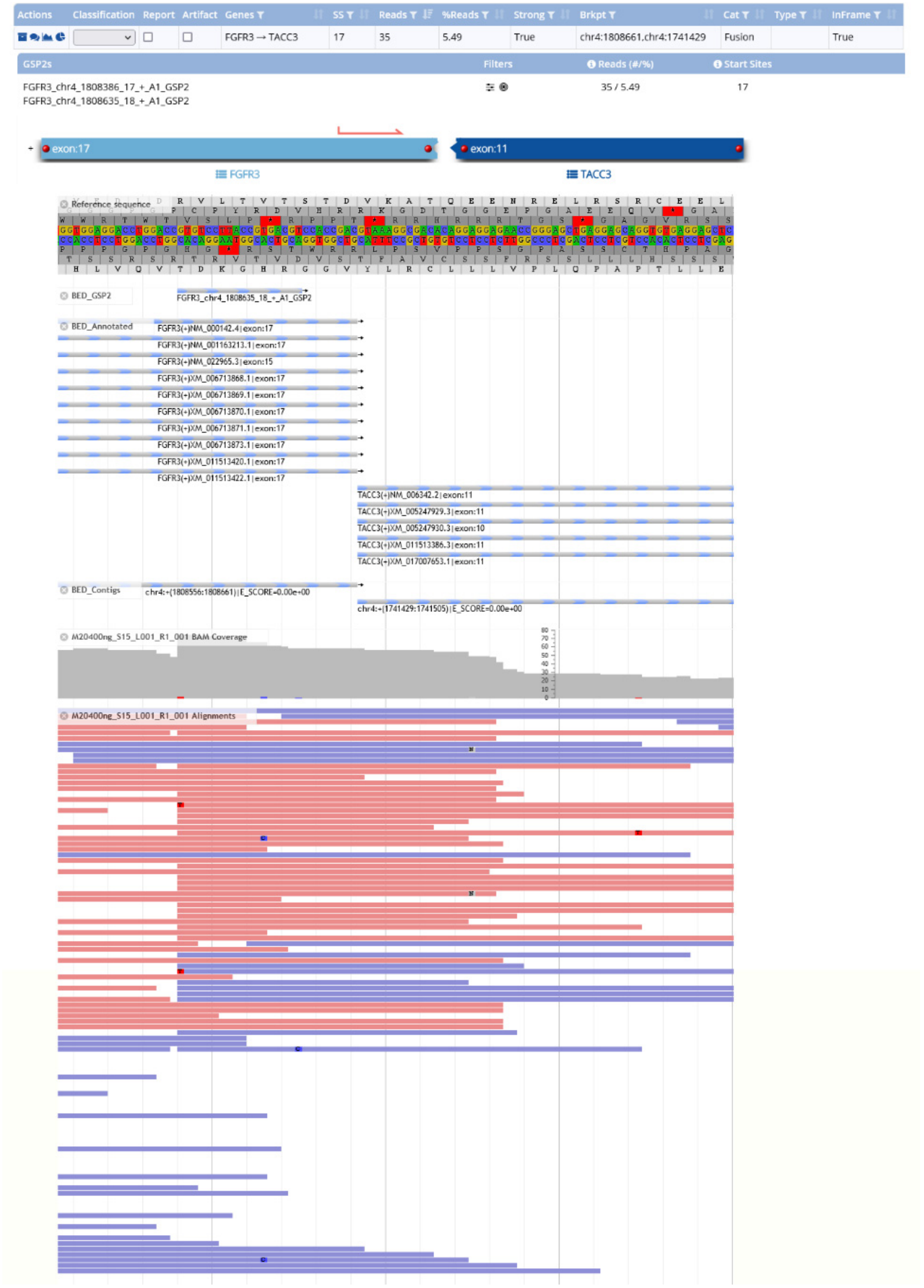

Figure S3. FGFR3::TACC3 fusion (M-20). FGFR3:NM_000142.4: exon:17; TACC3: NM_006342.2: exon:11.

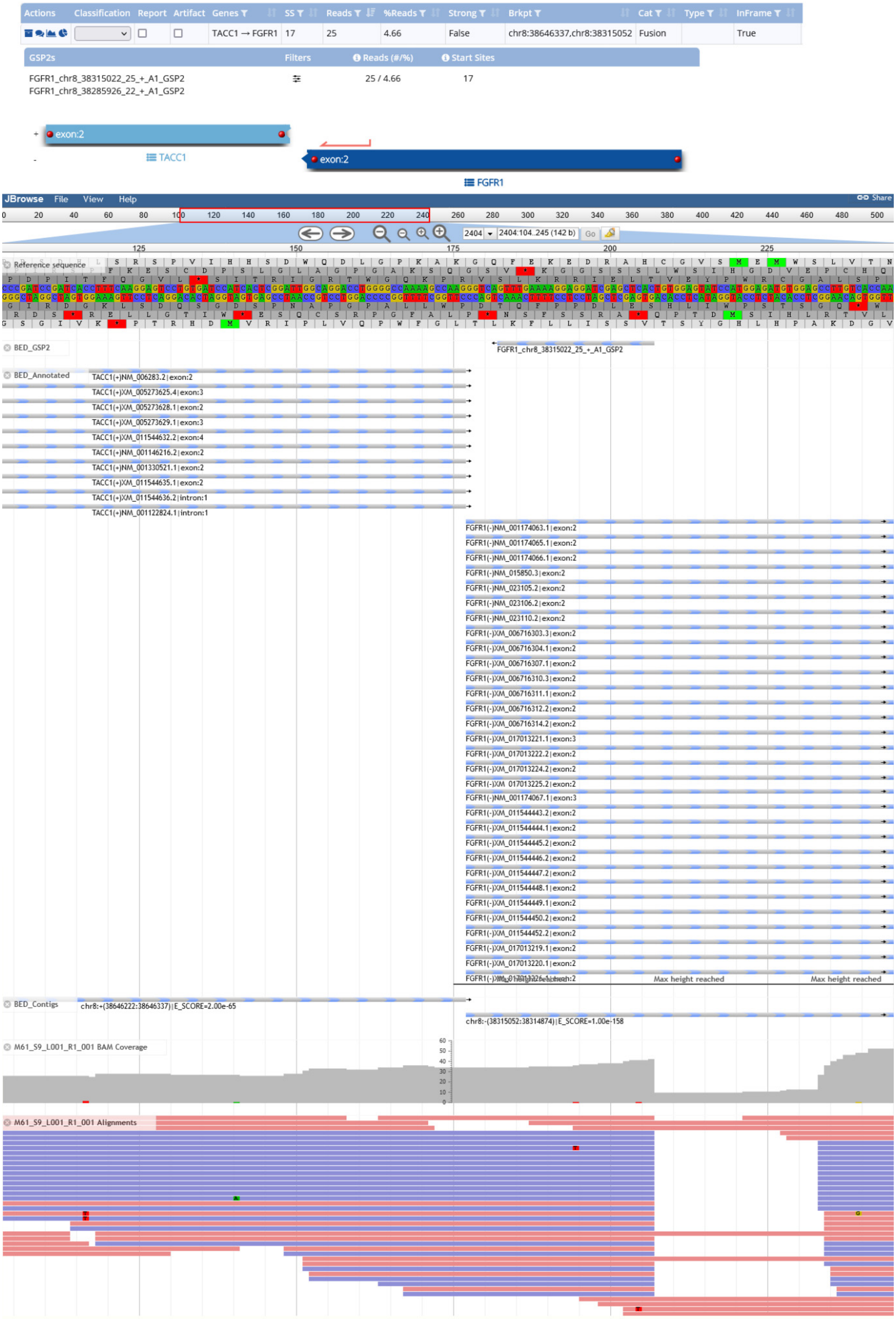

Figure S4. TACC1::FGFR1 fusion (M-61) TACC1: NM_006283.2:exon:2; FGFR1: NM_023110.3:exon:2.

Moes-Sosnowska, J.; Skupinska, M.; Lechowicz, U.; Szczepulska-Wojcik, E.; Skronska, P.; Rozy, A.; Stepniewska, A.; Langfort, R.; Rudzinski, P.; Orlowski, T.; et al. FGFR1–4 RNA-Based Gene Alteration and Expression Analysis in Squamous Non-Small Cell Lung Cancer. *Int. J. Mol. Sci.* 2022, *23*

Ranking Order (Better--Good--Average)

(a) Tumor tissue

Method/Ranking	1	2	3	4	5	6
Delta CT	ACTB	POLR2A	YWHAZ	HGPRT	B2M	GAPDH
BestKeeper	POLR2A	ACTB	YWHAZ	B2M	HGPRT	GAPDH
Normfinder	POLR2A	ACTB	YWHAZ	HGPRT	B2M	GAPDH
Genorm	POLR2A | ACTB		B2M	YWHAZ	HGPRT	GAPDH

(b) Tumor-adjacent tissue

Method/Ranking	1	2	3	4	5	6
Delta CT	ACTB	HGPRT	POLR2A	B2M	YWHAZ	GAPDH
BestKeeper	POLR2A	HGPRT	ACTB	YWHAZ	B2M	GAPDH
Normfinder	ACTB	HGPRT	POLR2A	B2M	YWHAZ	GAPDH
Genorm	YWHAZ | B2M		ACTB	POLR2A	HGPRT	GAPDH

(c) All samples

Method/Ranking	1	2	3	4	5	6
Delta CT	POLR2A	HGPRT	ACTB	YWHAZ	B2M	GAPDH
BestKeeper	POLR2A	ACTB	B2M	YWHAZ	HGPRT	GAPDH
Normfinder	POLR2A	HGPRT	ACTB	YWHAZ	B2M	GAPDH
Genorm	B2M | ACTB		POLR2A	HGPRT	YWHAZ	GAPDH

Recommended comprehensive ranking

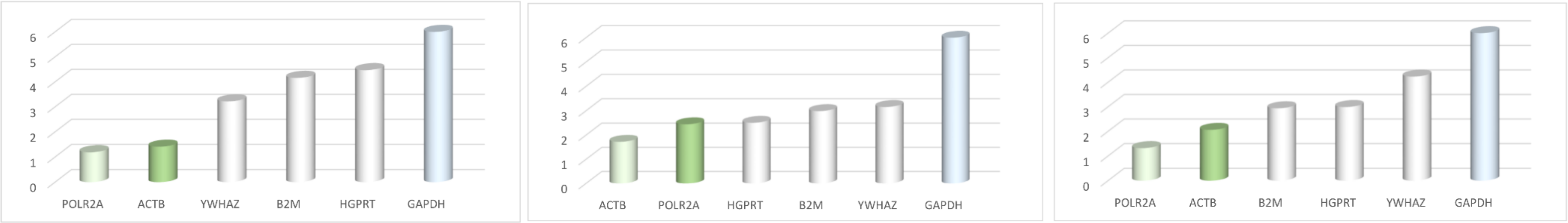

Figure S5. Expression stability ranking of six selected genes analyzed using RefFinder software. Results from GeNorm, Normfinder, BestKeeper, and ΔCT algorithms and summary of the results based on the four algorithms (from the most to the least stable expression) in (**a**) tumor tissue (n = 32); (**b**) tumor-adjacent normal tissue (n = 15); and (**c**) all analyzed samples (n = 47).
